# Insertion of [1.1.1]propellane into aromatic disulfides

**DOI:** 10.3762/bjoc.15.114

**Published:** 2019-05-28

**Authors:** Robin M Bär, Gregor Heinrich, Martin Nieger, Olaf Fuhr, Stefan Bräse

**Affiliations:** 1 Institute of Organic Chemistry, Karlsruhe Institute of Technology (KIT), Fritz-Haber-Weg 6, 76131 Karlsruhe, Germany; 2 Department of Chemistry, University of Helsinki, P.O. Box 55 (A. I. Virtasen aukio 1), 00014 University of Helsinki, Finland; 3 Institute of Nanotechnology (INT) and Karlsruhe Nano-Micro Facility (KNMF), Karlsruhe Institute of Technology (KIT), Hermann-von-Helmholtz-Platz 1, 76344 Eggenstein-Leopoldshafen, Germany; 4 Institute of Toxicology and Genetics, Karlsruhe Institute of Technology (KIT), Hermann-von-Helmholtz-Platz 1, 76344 Eggenstein-Leopoldshafen, Germany

**Keywords:** bicyclo[1.1.1]pentane, bioisosteres, disulfides, linkers, [1.1.1]propellane

## Abstract

Herein we present the synthesis of symmetrically and unsymmetrically substituted 1,3-bissulfanylbicyclo[1.1.1]pentanes from disulfides and [1.1.1]propellane. Bicyclo[1.1.1]pentanes (BCPs) recently gained interest as rigid linkers and as bioisosters of *para*-substituted benzene and alkyne moieties. The most promising precursor for BCPs is [1.1.1]propellane (**1**). The available methods to synthesize BCPs are quite limited and many groups contribute to the development of novel methods. The insertion of **1** into disulfide bonds is known, but has never been thoroughly investigated. In this study, we show that an UV initiated radical reaction can be used to synthesize symmetrically and unsymmetrically substituted BCP sulfides by reaction of [1.1.1]propellane (**1**) with disulfides. Depending on the ratio of **1** to the disulfide, only the BCP product (with up to 98% yield) or a mixture of BCP and [2]staffane can be obtained. The reaction tolerates functional groups such as halogens, alkyl and methoxy groups. The separation of the corresponding BCP and [2]staffane products is challenging but possible by column chromatography and preparative TLC in most cases. Single crystal X-ray diffraction analysis confirms the rod-like structure of the [2]staffanes that is often required in material applications.

## Introduction

**Scheme 1:**
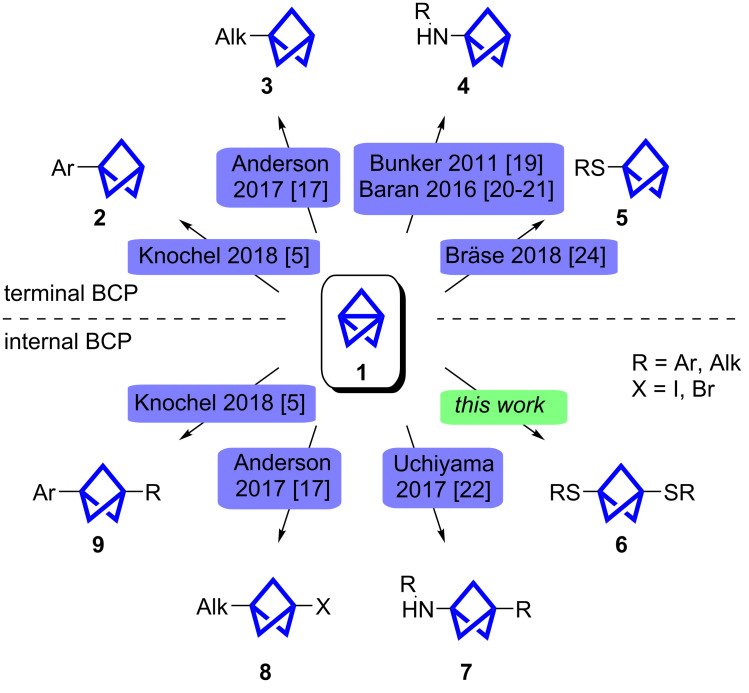
Summary of the most recent methods to obtain different BCPs from **1**.

Rigid structures are emerging in both materials science and medicinal chemistry [1]. Often referred to as bioisosteres, small strained hydrocarbons are used to replace phenyl [2–4] or alkyne groups [5] in well-known compounds, e.g., imatinib [3] and tazarotene [5]. The increase of the three-dimensionality and the disruption of the π-system can lead to improved properties, e.g., increased water-solubility of drug candidates [2] or the electronical separation of a photoswitch and a chromophore [6]. Often used moieties for these kinds of applications are triptycenes, cubanes, bicyclo[2.2.2]octanes (BCOs) and bicyclo[1.1.1]pentanes (BCPs) [1]. In their pioneering work Stepan et al. replaced a *para*-substituted fluorophenyl ring in the γ-secretase inhibitor BMS-708,163 with a BCP whereby the oral absorption and in vitro metabolic stability could be significantly increased [2]. BCPs are usually derived from [1.1.1]propellane (**1**) [7]. However, the available methods to synthesize useful BCP building blocks are quite limited and current research focuses on the development of such methods (Scheme 1).

After the first synthesis of [1.1.1]propellane (**1**) by Wiberg and Walker in 1982 [8] and the improved route by Szeimies et al. in 1985 [9] the compound and its reactions were intensely investigated [10]. Michl et al. synthesized terminally functionalized polymers derived from **1**, so-called [*n*]staffanes, and discussed their application as rigid tectones [11–12].

The characteristic reactivity of **1** emerges from the strained central bond, which was opened with free radicals in most cases. The reaction with diacetyl and subsequent oxidation leads to the important intermediate bicyclo[1.1.1]pentane-1,3-dicarboxylic acid which provides access to unsymmetrically substituted BCPs (not shown) [13]. It is assumed that the reaction of **1** with nucleophiles also proceeds through a radical mechanism [14–15]. The reaction with alkyl halides, initially described by Michl et al. [16], has recently been further investigated by Anderson et al. (Scheme 1) [17]. Both groups showed that the BCP halide can be lithiated to further modify the products. The opening of **1** with Grignard reagents enables the synthesis of aryl and some alkyl-substituted BCPs and a subsequent cross-coupling reaction [5,18].

To provide bicyclo[1.1.1]pentylamine as a building block in large-scale syntheses, Bunker et al. developed a synthesis of hydrazine BCP via a manganese-catalyzed reaction, which can easily be converted to the corresponding amine [19]. An alternative approach by Baran et al. installs the BCP late-stage at a secondary aliphatic amine via the corresponding turbo-amide [20–21]. Uchiyama et al. investigated the reaction mechanism of a radical multicomponent carboamination of **1**. This reaction provides access to internal BCPs next to amines [22].

The reaction of **1** with thiophenol has been known since 1985 and, given the high yield, used to determine the concentration of propellane solutions [23]. Our group investigated this reaction further and proved the generality of the thiol addition [24].

In analogy to this reaction, the insertion of **1** into disulfide bonds has been discovered early, but only very few examples can be found in the literature [10–11,25–28].

In this work, the insertion of [1.1.1]propellane (**1**) into disulfide bonds was investigated further to enable the synthesis of novel BCP building blocks. After optimizing the reaction conditions, the scope of the reaction was tested, followed by a proof-of-concept that enables a new route to unsymmetrically substituted BCPs.

## Results and Discussion

As a starting point, [1.1.1]propellane (**1**) has been prepared by a published procedure as a solution in diethyl ether [20], quantified by the reaction with thiophenol [23] and stored at −78 °C. Solutions of **1** were obtained with concentrations between 0.40–0.55 M.

### Optimization

**Scheme 2:**
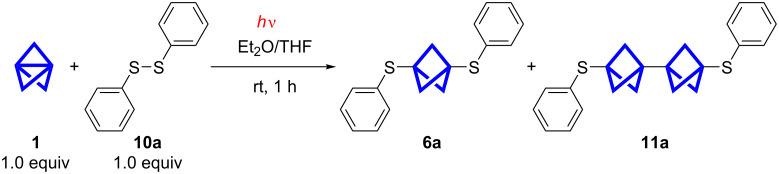
Screening reaction performed with different types of irradiation (see Figure 1).

**Figure 1:**
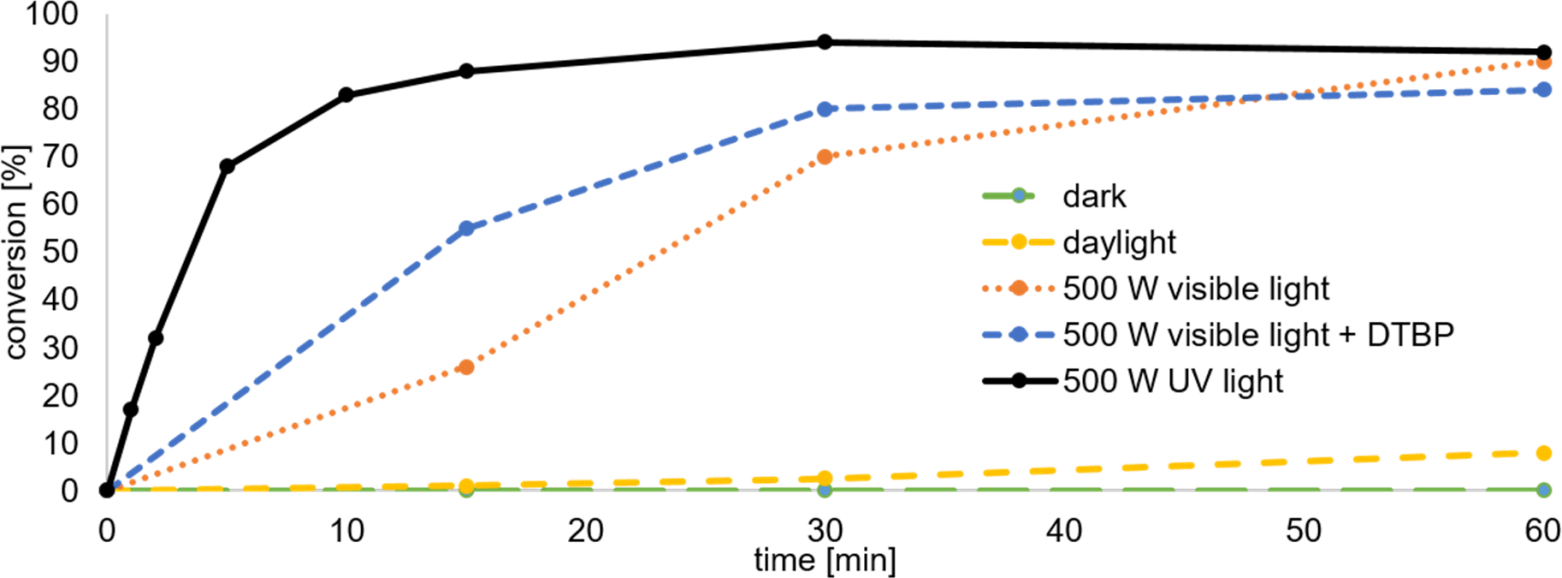
Optimization of the reaction conditions. The relative conversion was determined by GC–MS. The use of a radical initiator (di-*tert*-butyl peroxide, DTBP) led to increased amounts of insoluble polymer.

As some reactions of disulfides with **1** have been reported under different conditions we decided to compare and tune these conditions. Szeimies et al. used azobisisobutyronitrile (AIBN) as a radical initiator and heated the reaction mixture in a Carius tube to 80 °C [27]. Wiberg et al. initiated the reaction by irradiation with a 60 W light bulb overnight [10]. Michl et al. were the first to use UV irradiation in this reaction, but did not report detailed conditions [28]. They used diacetyldithiol and **1** to obtain bisacetylthio[*n*]staffanes. To find feasible conditions for the insertion of **1** into disulfide bonds, a screening with irradiations of different wavelengths and other reaction conditions was performed (Scheme 2). The screening reaction was set up with 1.0 equiv of diphenyl disulfide (**10a**) and 1.0 equiv of **1** in diethyl ether at room temperature and the consumption of the starting material was monitored by GC–MS over a period of 1 h (Figure 1). In this first screening approach the products were not isolated. The relative conversion was determined by integrating the signals of **10a** and the products **6a** and **11a**. No other signals were detected and the sum of the integrals was defined as 100%. This method cannot be used to determine yields or absolute concentrations as no internal standard was added.

In the dark, no conversion could be observed at all. The exposure to daylight led to a conversion of less than 10% after 1 h. With a 500 W halogen lamp, 90% of the starting material was consumed after 1 h. By the addition of di-*tert*-butyl peroxide (DTBP) as a radical initiator, the conversion could be accelerated in the beginning of the measurement. However, after the work-up of the reaction with radical initiator, an insoluble white solid, presumably longer [*n*]staffanes, was discovered. When the reaction was performed in a 500 W UV reactor (medium-pressure mercury-vapor lamps, 254 nm) without a radical initiator almost full conversion (≈90%) was observed after 15 min.

The use of UV light at room temperature seemed feasible and the conversion was faster than reported by Wiberg et al*.* [10]. The absence of a radical initiator is advantageous as the formation of longer [*n*]staffanes is usually promoted by the initiators. After this initial screening a reaction time of 20 min was chosen.

### Scope and limitations

The ratio of the products **6a** and **11a** could easily be changed by the amount of disulfide used in the reaction. We found that 3.0 equiv of the disulfide led to almost exclusive formation of **6a** (Table 1, entry 1). These conditions were applied to different aromatic disulfides and the corresponding products were obtained in fair to quantitative yields. The high yields for the BCPs **6** highlight the advantage of this mild method. Wiberg et al. reported a yield of 45% for **6a** [10] and Szeimies et al. obtained 63% of **6a** and 27% of **11a** [27]. Halogen substitutions were tolerated as well as methyl and methoxy groups. All products of substituted aromatic disulfides are previously unreported.

**Table 1:** Results of the insertion of **1** into aromatic disulfide bonds. The ratio of **1**:**10** determines the amount of [2]staffane formed.

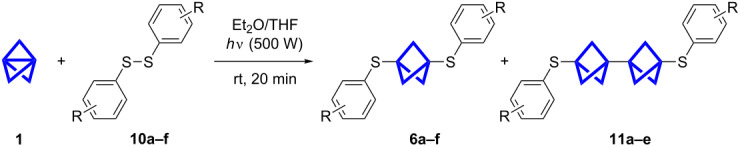					

Entry	Disulfide **10**	R	Ratio of **1**:**10**	Yield **6** [%]	Yield **11** [%]

1	**10a**	H	1:3	98^a^	–^b^
2	**10a**	H	1:1	51^c^	5^c^
3	**10a**	H	2:1	32^c^	10^c^
4	**10a**	H	3:1	33^c^	20^c^
5	**10b**	4-Cl	1:3	98^a^	–^b^
6	**10b**	4-Cl	2:1	34^c^	15^c^
7	**10c**	3,5-Cl_2_	1:3	96^a^	–^b^
8	**10c**	3,5-Cl_2_	2:1	34^c^	8^c^
9	**10d**	4-Me	1:3	96^a^	traces
10	**10d**	4-Me	2:1	35^c^	12^c^
11	**10e**	4-OMe	1:3	94^a^	traces
12	**10f**	2-Ph	1:3	61^a^	–^b^

^a^Isolated yield, purified by column chromatography. ^b^Not observed. ^c^Isolated yield, purified by preparative TLC.

**Figure 2:**
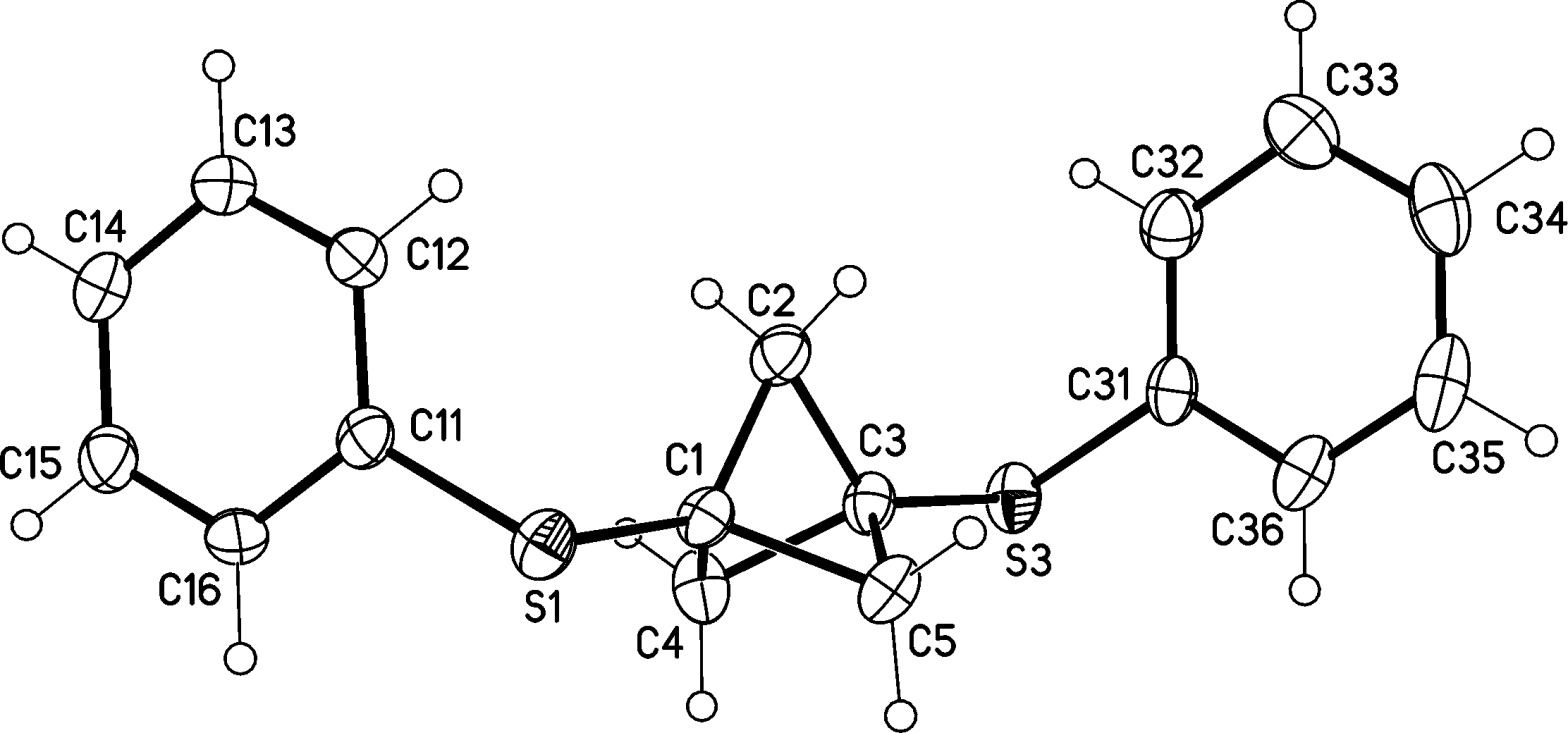
Molecular structure of **6a** (displacement parameters are drawn at 50% probability level), distance C1–C3 1.844(3) Å.

The analysis of compound **6a** by single-crystal X-ray diffraction [29] revealed a distance of 1.844(3) Å between the bridgehead carbons of the BCP unit (Figure 2).

When 2.0 equiv of **1** and 1.0 equiv of **10a** were used, the yield of **6a** dropped dramatically and the [2]staffane product **11a** was obtained in poor yield (Table 1, entry 3). The separation was possible by preparative TLC. It is assumed that longer staffane chains are formed in this reaction. However, the isolation and characterization of these compounds was not successful. The influence of the ratio of **1**:**10** on the formation of the [2]staffane was also investigated for some of the substituted disulfides. The overall trend was the same for all reactions with yields of around 35% for **6** and between 8–15% for **11**. The yield of **11a** could be increased to 20% by using a ratio of 3:1 (Table 1, entry 4).

**Scheme 3:**
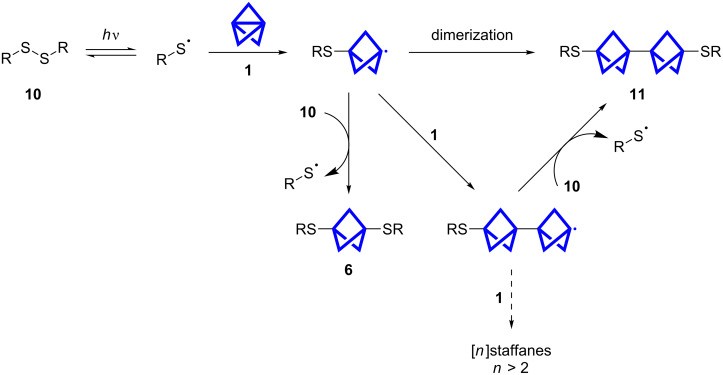
Proposed mechanism of the propellane insertion into disulfide bonds.

The radical reaction as proposed in Scheme 3 should lead to higher yields of **11** with an increasing amount of **1**. As the kinetically controlled reactions depend on the concentration of the reaction partners, the dimerization becomes less dominant with a low concentration of the BCP radical. The propagation with the disulfide proceeds faster as the disulfide concentration is higher. The formation of staffanes seems to be less favored than the propagation with the disulfide. This observation is in accordance with previous calculations and experimental results [17,22].

### Insertion into aliphatic disulfides

**Scheme 4:**
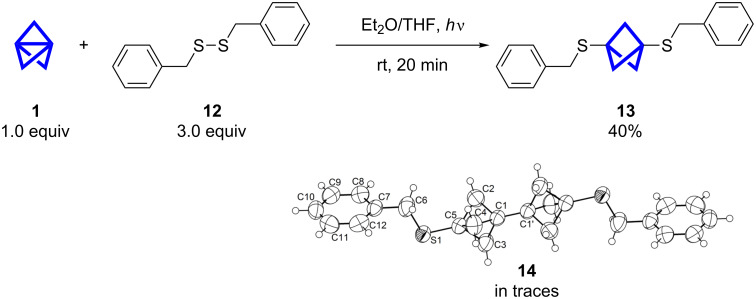
The insertion of **1** into dibenzyl disulfide (**12**) led to the formation of BCP **13** and traces of [2]staffane **14**. The structure of **14** could be proven by single-crystal X-ray diffraction (displacement parameters are drawn at 50% probability level).

To investigate the insertion of **1** into aliphatic disulfides, benzyl disulfide **12** was chosen as a model compound. As the formation of thiyl radicals is critical to the reaction and most aliphatic disulfides do not absorb UV light at the used wavelength, radical initiators should be taken into account for other disulfides. However, for **12** the reaction was successful without radical initiator and the BCP **13** was obtained as the main product (Scheme 4). The purification was possible by HPLC (reversed-phase) and the yield was significantly lower compared to the aromatic disulfides. Szeimies et al. also synthesized alkyl-substituted BCP and staffane sulfides with the previously described method (see optimization) in similar yields [27]. Presumably, there are two factors contributing to the lowered yield. The bond dissociation energy (BDE) of dialkyldisulfides is higher than the BDE of diaryl disulfides [30]. Therefore lower concentrations of thiyl radicals are present to initiate the reaction. The second reason could be the absorption of the UV irradiation. In the benzyl group only the aromatic part can absorb the light and a transfer has to take place to promote the homolytic cleavage of the disulfide.

In first purification attempts [2]staffane **14** could be identified in a mixture with **13** in traces. From the product mixture we could obtain single-crystals of **14** to confirm the structure by X-ray diffraction (Scheme 4) [31]. The rod-like structure of the [2]staffane unit now becomes more tangible. The distance in one BCP unit equals 1.857(3) Å (C1–C5) and the two units are 1.491(4) Å apart from each other (C1–C1’).

### Reaction with different mixtures of disulfides

**Scheme 5:**
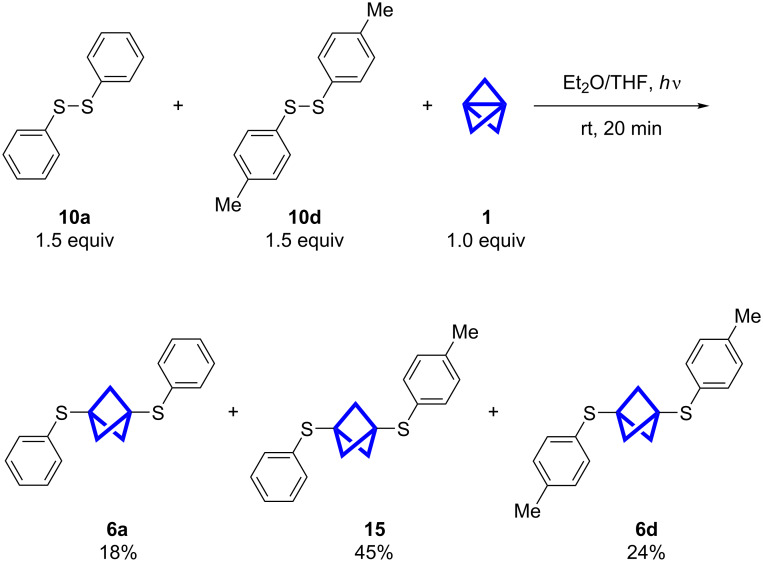
Reaction of propellane (**1**) with the two disulfides **10a** and **10d**. When two different disulfides were used, all three possible products were obtained. The yields were determined by NMR spectroscopy (Figure 3).

**Figure 3:**
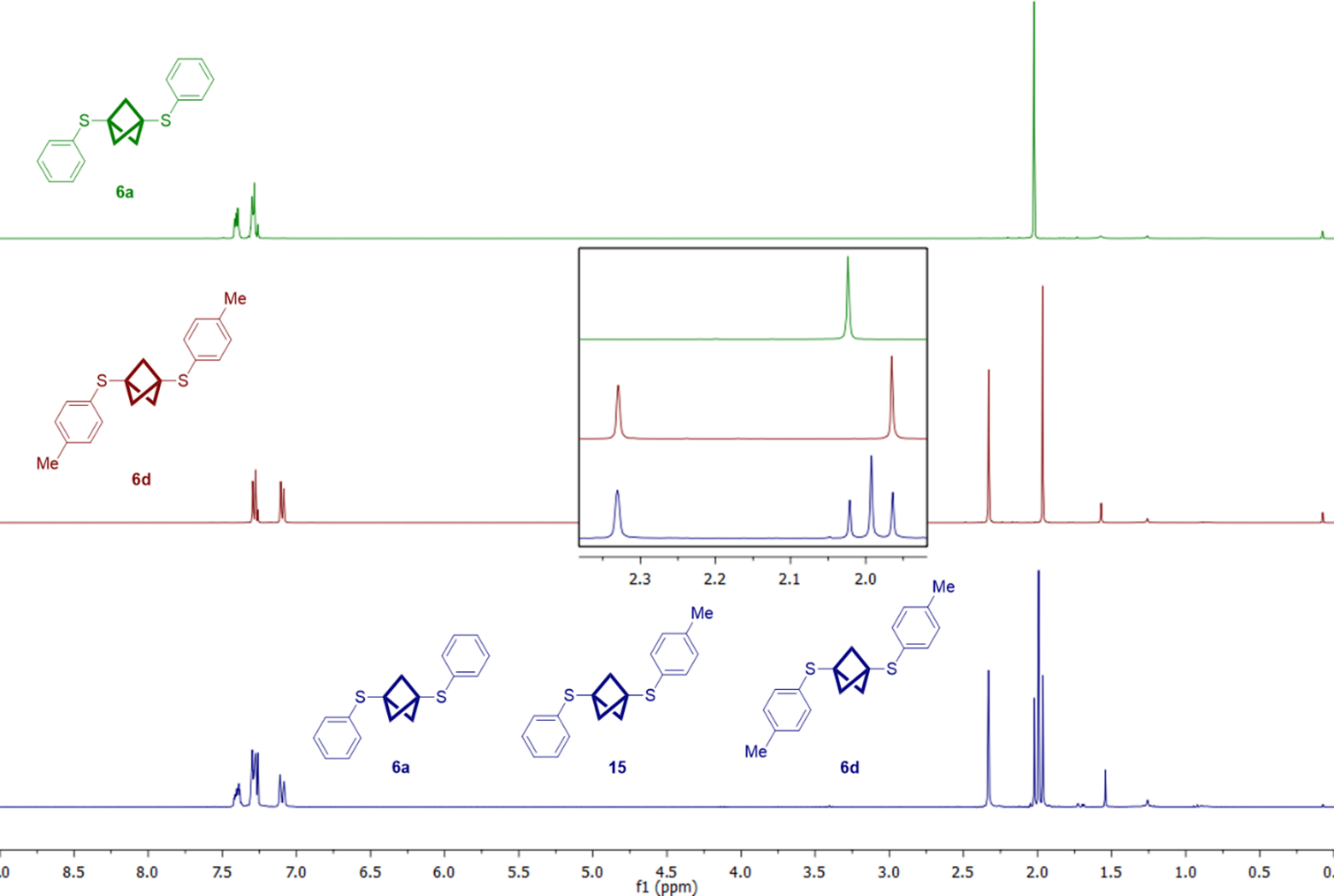
NMR spectra of pure **6a** (green) and **6d** (red) and the obtained mixture with the new compound **15** (blue).

The synthesis of useful unsymmetrically substituted BCPs remains a challenge and only few direct routes from **1** are available (see Introduction). The insertion into disulfides could provide an alternative approach. With the two disulfides **10a** and **10d** as starting materials a product mixture of **6a**, **15** and **6d** was obtained (Scheme 5). The yields were determined by NMR spectroscopy as the products could not be separated by column chromatography (Figure 3). Analytical samples could be obtained by purification of this mixture via HPLC (reversed-phase column). If one assumes equal reaction rates for the propellane insertion, independent from the substitution of the disulfide, a product mixture with the ratio 1:2:1 would be expected. The obtained yields of 18, 45 and 24%, respectively differ slightly from this ratio.

**Scheme 6:**
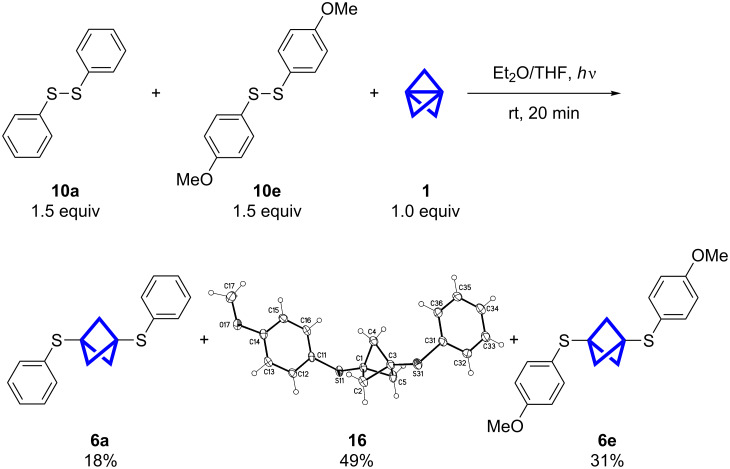
The reaction of **1** with the two disulfides **10a** and **10e** led to the known products **6a**, **6e** and to the unsymmetrically substituted BCP **16** as the main product. The compounds could be separated by column chromatography and preparative TLC. The structure of **16** could be confirmed by single-crystal X-ray diffraction (displacement parameters are drawn at 50% probability level).

To facilitate the separation of the products we switched the disulfides used in this reaction to **10a** and **10e** (Scheme 6). With the bigger difference in polarity of the substituents, the products **6a**, **16** and **6e** could be separated by column chromatography and preparative TLC. Again, we observed a deviation from the expected product ratio. This leads to the assumption that the 4-methoxybenzenethiyl radical is either formed faster and/or reacts more rapidly with **1** than the corresponding benzenethiyl radical. The calculated BDE (DFT calculation) of **10e** is slightly lower with 49.0 kcal/mol compared to **10a** with 54.5 kcal/mol [32]. This difference hints towards the faster formation of the 4-methoxybenzenethiyl radical, but the values are in the same region. To ascertain the main factor for this trend further investigations are necessary. We observed the same trend in the thiol addition to **1**, when thiophenol and 4-methoxythiophenol were used in a competitive reaction [24].

The structure of **16** could be confirmed by single-crystal X-ray diffraction [33]. With **16** as the main product of this reaction, this method provides new access to unsymmetrically substituted BCPs directly from **1** and purely symmetrical starting materials. This finding will be pursued with a selective cleavage or oxidation of one sulfide in further studies.

## Conclusion

The reported reaction of [1.1.1]propellane with aromatic and to a certain extend aliphatic disulfides provides access to [2]staffanes and symmetrical and unsymmetrical BCPs. The separation of these compounds is often challenging due to the similar polarity. However, the reaction conditions can be tuned to obtain solely the BCP compound in high yields. By mixing two aromatic disulfides in this reaction three different products can be obtained, with the unsymmetrically substituted compound as the main product. This method will become particularly interesting if the two sulfides of the product can be modified individually. This approach to unsymmetrically substituted BCPs will be further investigated.

## Supporting Information

Full experimental details and analytical data (^1^H NMR, ^13^C NMR, X-ray analysis) are provided in the following files.

File 1 Description and analyses.

File 2 Spectra.

File 3 RInChIs.

File 4 DOIs data repository.

File 5 Reference samples molecule archive.
