# Supplementary material for: Insertion of [1.1.1]propellane into aromatic disulfides
Source: Beilstein J Org Chem. 2019 May 28;15:1172–80. doi: 10.3762/bjoc.15.114 (PMC6604700; doi:10.3762/bjoc.15.114)
Supplement: File 1 — Description and analyses. [file Beilstein_J_Org_Chem-15-1172-s001.pdf]

BEILSTEIN JOURNAL OF ORGANIC CHEMISTRY

Supporting Information

for

Insertion of [1.1.1]propellane into aromatic disulfides

Robin M. Bär, Gregor Heinrich, Martin Nieger, Olaf Fuhr and Stefan Bräse

Beilstein J. Org. Chem. **2019,** *15,* 1172–1180. doi:10.3762/bjoc.15.114

Description and analyses

License and Terms: This is a supporting information file under the terms of the Creative Commons Attribution License (http://creativecommons.org/licenses/by/4.0). Please note that the reuse, redistribution and reproduction in particular requires that the authors and source are credited.
The license is subject to the *Beilstein Journal of Organic Chemistry* terms and conditions: (https://www.beilstein-journals.org/bjoc)

Contents

Readme S2
1 Versions S2
2 General remarks S2
3 Optimization experiments S3
4 General procedures S4
5 Synthesis S4
6 Crystallographic information S23

Readme

This Supplemental Information was created automatically by an export function of the Software Chemotion-ELN (ELN = electronic lab notebook) and the listed information was retrieved via the electronic lab notebook. Some changes were made to the original export document to improve the readability. Some information differs to standard descriptions in other Supporting Information and are therefore explained here:
(1) We introduce a novel manner to label molecules. The labeling in the form of {A|**1a**} refers, indicated by the "A" to a more general sorting of the molecule according to its function in the General Procedure. The listing of starting materials and reagents (e.g., A, B), solvents (S1, S2..) and products (P1, P2) is given for all general procedures, allowing the labeling of the molecules in the single reaction according to the label given in the general procedure.
The common alpha numeric label which refers to the unique number of the molecules as given in the article is added in addition as usual in bold after the slash "|**1a**".
(2) We added general identifiers and properties which can be calculated with a given molecular structure at the beginning of each analysis of the molecules. This procedure should facilitate the proofreading and the identification of the molecules (automatically generated).

1 Versions

We used OpenBabel Version 2.4.1 including InChI 1.04 for the generation of the given identifiers. SMILES are given according to Daylight.

2 General remarks

^{1}H NMR spectra were recorded on BRUKER Avance AV 300 (300 MHz), BRUKER Avance 400 (400 MHz) and BRUKER Avance DRX 500 (500 MHz) spectrometers. Chemical shifts are given in parts per million (δ/ppm), downfield from tetramethylsilane (TMS) are referenced to chloroform (7.26 ppm) as internal standard. All coupling constants are absolute values and *J* values are expressed in Hertz (Hz). The description of signals include: s = singlet, br. s = broad singlet, d = doublet, bd = broad doublet, t = triplet, dd = doublet of doublets, ddd = doublet of doublet of doublets, dt = doublet of triplets, q = quartet, quin = quintet, sxt = sextet, sept = septet, m = multiplet. The spectra were analyzed according to first order.
^{13}C NMR spectra were recorded on BRUKER Avance 400 (100 MHz) and BRUKER Avance DRX 500 (125 MHz) spectrometers. Chemical shifts are expressed in parts per million (δ/ppm) downfield from tetramethylsilane (TMS) and are referenced to chloroform (77.16 ppm) as internal standard.
MS (EI) (electron impact mass spectrometry): Finnigan MAT 95 (70 eV). The molecular fragments are quoted as the relation between mass and charge (*m*/*z*), the intensities as a percentage value relative to the intensity of the base signal (100%).
HRMS: all HRMS data are recorded with the Finnigan MAT 95 (EI-method).

GC–MS (gas chromatography – mass spectrometry): Agilent GC 6890N, MS 5975B VL MSD, column Agilent HP-5MS, helium as carrier gas, EI as ionization method.
IR (infrared spectroscopy): ATR spectra were recorded by diamond crystal on Bruker ALPHA-IR.
Melting points were detected on an OptiMelt MPA100 device from the company *Stanford Research System*.
HPLC (high-performance liquid chromatography): VDSpher 100 C18-E, 250 × 20 mm, 5 µm, eluent: water/acetonitrile, flow: 16 mL/min.
Routine monitoring of reactions were performed using silica gel coated aluminium plates (Merck, silica gel 60, F254) which were analyzed under UV-light at 254 nm and/or dipped into a solution of Seebach reagent (2.5% phosphor molybdic acid, 1.0% cerium(IV) sulfate tetrahydrate and 6.0% sulfuric acid in H_2O, dipping solution) and heated with a heat gun. Solvent mixtures are understood as v/v.
Solvents, reagents and chemicals were purchased from Sigma-Aldrich, Alfa Aesar, ABCR, Thermo Fisher Scientific, TCI and VWR and used without further purification unless stated otherwise.
The following disulfides were purchased from the mentioned companies: **10a–e**, **12**.
The disulfide **10f** was synthesized as described below.

3 Optimization experiments

For the optimization experiment, the following reaction was set up under different conditions and a sample was taken with a syringe after different times. The samples were diluted with ethyl acetate (1.5 mL) and checked by GC–MS. The relative conversion was calculated based on the integrals of the signals of **10a**, **6a** and **11a**, respectively. There were no additional peaks visible in the spectrum.

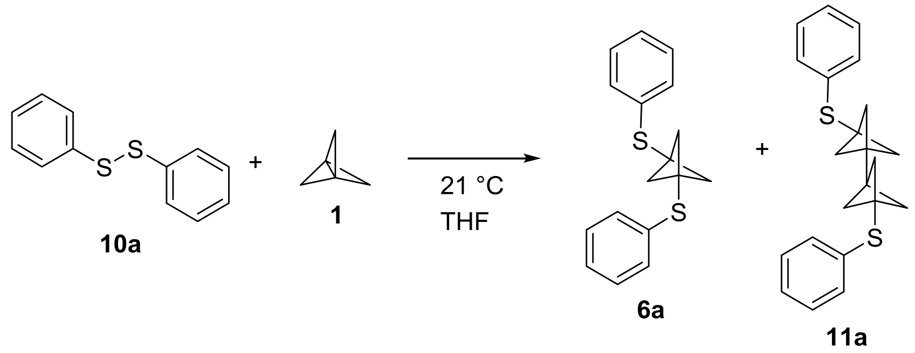

The propellane solution {B|**1**} (0.0345 g, 0.522 mmol, 1.00 equiv) was added to a solution of diphenyl disulfide {A|**10a**} (0.114 g, 0.523 mmol, 1.00 equiv) in THF {S1} (1.0 mL) under argon atmosphere in a quartz flask and the mixture was irradiated with different light sources for 2 h.

Table 1: Relative conversion of the disulfide **10a** determined by GC–MS.

**time [min]**	**0**	**1**	**2**	**5**	**10**	**15**	**30**	**60**	**90**	**120**
dark	0%					0%	0%	0%	3%	7%
daylight	0%					1%	2.5%	8%	19%	33%
500 W visible light	0%					26%	70%	90%	96%	95%
500 W visible light	0%					55%	80%	84%	87%	93%

+ 20 mol% DTBP									
500 W UV light	0%	17%	32%	68%	83%	88%	94%	92%	97%	98%

4 General procedures

4.1 General procedure a

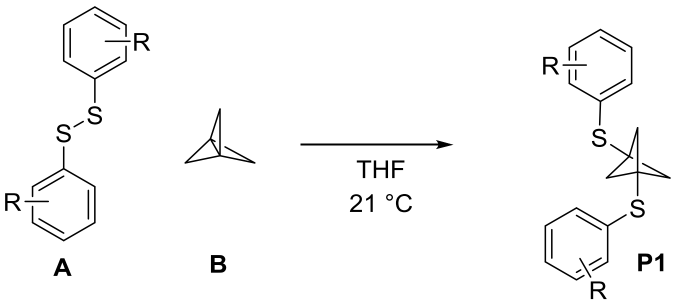

The propellane solution (1.00 equiv in Et_2O) was added to a solution of the disulfide (3.00 equiv) in THF under argon atmosphere in a quartz flask and the mixture was irradiated with UV light (500 W) for 20 min. After evaporation of the solvent, the crude residue was purified *via* column chromatography or preparative TLC.

4.2 General procedure b

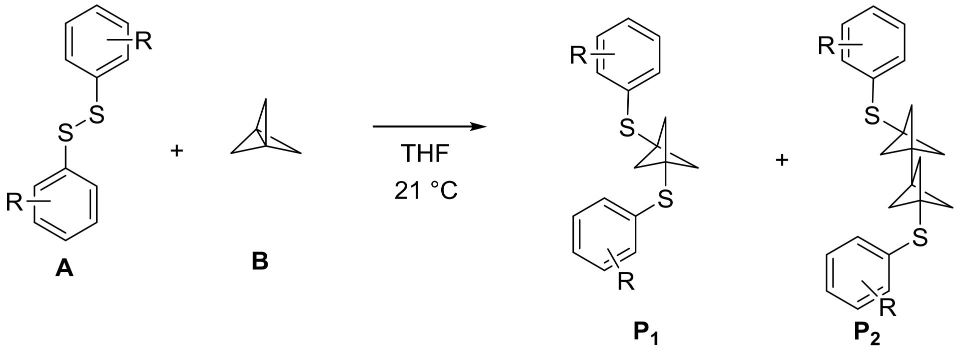

The propellane solution (2.00 equiv in Et_2O) was added to a solution of the disulfide (1.00 equiv) in THF under argon atmosphere in a quartz flask and the mixture was irradiated with UV light (500 W) for 20 min. After evaporation of the solvent, the crude residue was purified *via* preparative TLC.

5 Synthesis

5.1 [1.1.1]Propellane (**1**)

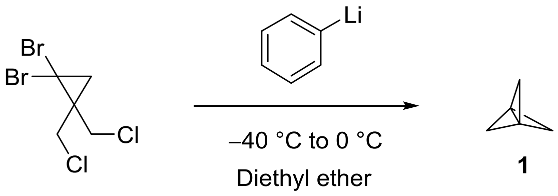

Name {P1|**1**}: [1.1.1]Propellane; Formula: C_5H_6; Molecular Mass: 66.1011; Exact Mass: 66.0470; Smiles: C1C23C1(C2)C3; InChIKey: ZTXSPLGEGCABFL-UHFFFAOYSA-N

[1.1.1]Propellane (**1**): In a flame-dried round-bottomed flask that has been purged with argon 1,1-dibromo-2,2-bis(chloromethyl)cyclopropane (4.04 g, 12 mmol, 1.00 equiv) was dissolved in diethyl ether (17.0 mL) and cooled to −40 °C. 1.9 M Phenyllithium solution (2.06 g, 12.9 mL, 25 mmol, 2.00 equiv) in dibutyl ether was added dropwise under vigorous stirring. After complete addition the mixture was allowed to warm to 0 °C and stirred at this temperature for further 2 h. The reaction flask was attached to an argon purged rotavap with dry ice condenser. The receiving flask was cooled to −78 °C and the product was distilled together with diethyl ether. The water bath was set to 20 °C and the pressure was reduced from 500 mbar to 20 mbar slowly. A solution of **1** in diethyl ether was obtained and stored at −78 °C. To quantify the concentration of **1** an aliquot was taken and reacted with thiophenol (see **5a**). With the calculated concentration the yield could be determined retrospectively and ranged from 57% (0.40 M) to 78% (0.55 M) in different batches.

5.2 3-Phenylsulfanylbicyclo[1.1.1]pentane (**5a**)

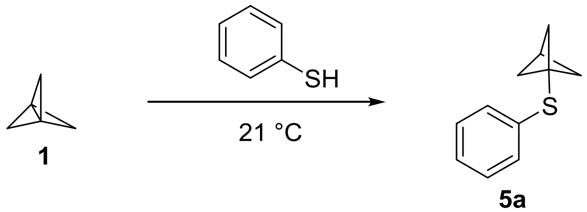

1 **5a**

Name {P1|**5a**}: 3-Phenylsulfanylbicyclo[1.1.1]pentane; Formula: $C_{11}H_{12}S$; Molecular Mass: 176.2780; Exact Mass: 176.0660; Smiles: c1ccc(cc1)SC12CC(C1)C2; InChIKey: AXGWEHWIGICNLX-UHFFFAOYSA-N

[1.1.1]Propellane (**1**) quantification: A 1 M thiophenol solution in diethyl ether (165 mg, 1.50 mL, 1.5 mmol) was added to 1 mL of the propellane (**1**) solution and the mixture was stirred for 15 min at rt. The solution was diluted with 10 mL of pentane and washed with 10 mL of 1 M NaOH solution. The organic layer was dried by the addition of Na_2SO_4. The mixture was filtered through a glass funnel and the solvent was evaporated under reduced pressure to give the target compound **5a** (0.0920 g, 0.522 mmol) as a colorless oil.
The yield of the reaction is assumed to be quantitative according to K. B. Wiberg, S. T. Waddell, *J. Am. Chem. Soc.* **1990**, *112*, 2194–2216.

^{1}H NMR (300 MHz, $CDCl_3$, ppm) δ = 7.47–7.42 (m, 2H, Ar-H), 7.34–7.27 (m, 3H, Ar-H), 2.72 (s, 1H, C*H*), 1.96 (s, 6H, 3xCH_2). ^{13}C NMR (100 MHz, $CDCl_3$, ppm) δ = 134.2 (C_{quat}, C_{Ar}S), 133.6 (+, 2xCH_{Ar}), 128.9 (+, 2xCH_{Ar}), 127.6 (+, CH_{Ar}), 54.1 (–, 3xCH_2), 45.8 (C_{quat}, CCH_2), 28.8 (+, *C*H).
In accordance with R. M. Bär, S. Kirschner, M. Nieger, S. Bräse, *Chem. Eur. J.* **2018**, *24*, 1373.

The conditions for this reaction were deposited at Chemotion-repository:

https://dx.doi.org/10.14272/reaction/SA-FUHFF-UHFFFADPSC-AXGWEHWIGI-UHFFFADPSC-NUHFF-NUHFF-NUHFF-ZZZ

Original Data for the target compound can be accessed on the repository Chemotion:

**Type**	**DOI**	**Owner**[1]
**5a**	10.14272/AXGWEHWIGICNLX-UHFFFAOYSA	
1H NMR	https://dx.doi.org/10.14272/AXGWEHWIGICNLX-UHFFFAOYSA-N/1HNMR	A
13C NMR	https://dx.doi.org/10.14272/AXGWEHWIGICNLX-UHFFFAOYSA-N/13CNMR	A

[1] A = data of the authors; B = data provided by others

5.3 1,3-Bis(phenylthio)bicyclo[1.1.1]pentane (**6a**)

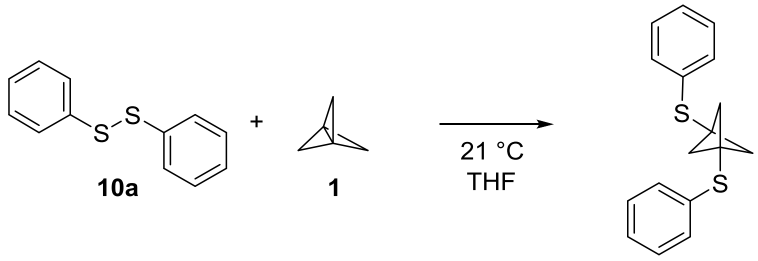

10a **1** **6a**

Name {P1|**6a**}: 1,3-Bis(phenylthio)bicyclo[1.1.1]pentane; Formula: $C_{17}H_{16}S_2$; Molecular Mass: 284.4389; Exact Mass: 284.0693;
Smiles: c1ccc(cc1)SC12CC(C1)(C2)Sc1ccccc1;
InChIKey: GHXRODNNSAZUSG-UHFFFAOYSA-N

According to General Procedure a: {A|**10a**} Diphenyl disulfide (0.342 g, 1.57 mmol, 3.00 equiv); {B|**1**} [1.1.1]Propellane (0.0345 g, 0.522 mmol, 1.00 equiv); {S1} Tetrahydrofuran (1.0 mL); Yield {P1|**6a**} = 98% (0.145 g, 0.510 mmol).
The obtained crude product was purified *via* flash-chromatography on silica gel using pentane to pentane/diethyl ether 100:1. The product was obtained as a white solid. m.p. 54 °C. R_f = 0.19 (pentane).
^{1}H NMR (400 MHz, $CDCl_3$, ppm) δ = 7.42–7.39 (m, 4H, Ar-H), 7.31–7.28 (m, 6H, Ar-H), 2.02 (s, 6H, 3xCH_2); ^{13}C NMR (100 MHz, $CDCl_3$, ppm) δ = 134.0 (+, 4xCH_{Ar}), 133.3 (C_{quat}, 2xC_{Ar}S), 129.0 (+, 4xCH_{Ar}), 128.1 (+, 2xCH_{Ar}), 57.5 (–, 3xCH_2), 42.7 (C_{quat}, 2xCCH_2); EI (***m/z***, 70 eV, 50 °C): 284 (3) $[M]^+$, 175 (100) $[M–C_6H_5S]^+$, 109 (13) $[C_6H_5S]^+$; HRMS (EI, 70 eV): calcd for $C_{17}H_{16}{}^{32}S_2$ $[M]^+$: 284.0693, found 284.0692; IR (ATR, ṽ) = 3055, 2989, 2961, 2925, 2907, 2867, 1951, 1878, 1715, 1578, 1572, 1472, 1436, 1388, 1327, 1300, 1197, 1159, 1126, 1099, 1088, 1062, 1020, 1000, 987, 928, 907, 890, 849, 759, 739, 703, 687, 554, 507, 476, 422, 408 cm^{-1}.

The conditions for this reaction were deposited at Chemotion-repository:

https://dx.doi.org/10.14272/reaction/SA-FUHFF-UHFFFADPSC-GHXRODNNSA-UHFFFADPSC-NUHFF-NUHFF-NUHFF-ZZZ

Original Data for the target compound can be accessed on the repository Chemotion:

**Type**	**DOI**	**Owner**[1]
**6a**	GHXRODNNSAZUSG-UHFFFAOYSA-N	
1H NMR	https://dx.doi.org/10.14272/GHXRODNNSAZUSG-UHFFFAOYSA-N/1HNMR	A
13C NMR	https://dx.doi.org/10.14272/GHXRODNNSAZUSG-UHFFFAOYSA-N/13CNMR	A
Mass	https://dx.doi.org/10.14272/GHXRODNNSAZUSG-UHFFFAOYSA-N/Mass	A
HRMS	https://dx.doi.org/10.14272/GHXRODNNSAZUSG-UHFFFAOYSA-N/Mass.1	A

IR	https://dx.doi.org/10.14272/GHXRODNNSAZUSG-UHFFFAOYSA-N/IR	A
X-Ray	https://dx.doi.org/10.14272/GHXRODNNSAZUSG-UHFFFAOYSA-N/Crystal-Structure	A

[1] A = data of the authors; B = data provided by others

5.4 1,3-Bis((4-chlorophenyl)thio)bicyclo[1.1.1]pentane (**6b**)

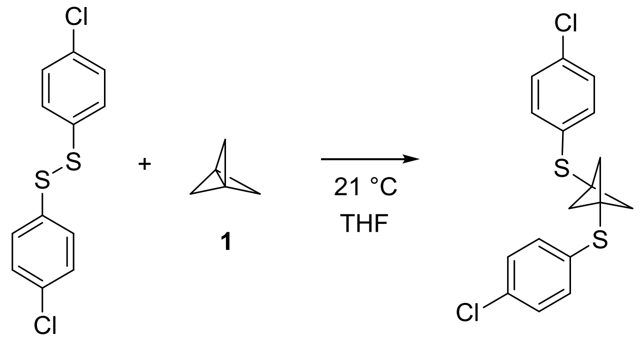

Name {P1|**6b**}: 1,3-Bis((4-chlorophenyl)thio)bicyclo[1.1.1]pentane; Formula: $C_{17}H_{14}Cl_2S_2$; Molecular Mass: 353.3291; Exact Mass: 351.9914;
Smiles: Clc1ccc(cc1)SC12CC(C1)(C2)Sc1ccc(cc1)Cl;
InChIKey: SJOKHIZPCGZHAT-UHFFFAOYSA-N

According to General Procedure a: {A|**10b**} Bis(4-chlorophenyl) disulfide (0.411 g, 1.43 mmol, 3.00 equiv); {B|**1**} [1.1.1]Propellane (0.0315 g, 0.476 mmol, 1.00 equiv); {S1} Tetrahydrofuran (1.0 mL); Yield {P1|**6b**} = 98% (0.165 g, 0.467 mmol).
The obtained crude product was purified *via* flash-chromatography on silica gel using pentane. The product was obtained as a white solid. m.p. 94 °C. R_f = 0.38 (pentane). ^{1}H NMR (300 MHz, $CDCl_3$, ppm) δ = 7.35–7.27 (m, 8H, Ar-H), 1.99 (s, 6H, 3xCH_2); ^{13}C NMR (100 MHz, $CDCl_3$, ppm) δ = 135.4 (+, 4xCH_{Ar}), 134.5 (C_{quat}, 2xC_{Ar}Cl), 131.6 (C_{quat}, 2xC_{Ar}S), 129.3 (+, 4xCH_{Ar}), 57.4 (+, 3xCH_2), 42.7 (C_{quat}, 2xCCH_2); EI (***m/z***, 70 eV, 90 °C): 356/354/352 (1/2/3) $[M]^+$, 211/209 (34/100) $[M–C_6H_4ClS]^+$, 174 (13) $[M–C_6H_4ClS–Cl]^+$, 145/143 (5/12) $[C_6H_4ClS]^+$, 108 (10) $[C_6H_4S]^+$; HRMS (EI, 70 eV): Calcd for $C_{17}H_{14}{}^{35}Cl_2{}^{32}S_2$ $[M]^+$: 351.9916, found 351.9914; IR (ATR, ṽ) = 382, 425, 476, 509, 544, 557, 703, 744, 761, 776, 817, 892, 925, 1010, 1089, 1133, 1171, 1196, 1262, 1289, 1384, 1443, 1473, 1572, 1639, 1740, 1897, 2854, 2868, 2907, 2924, 2959, 2987 cm^{-1}.

The conditions for this reaction were deposited at Chemotion-repository:

https://dx.doi.org/10.14272/reaction/SA-FUHFF-UHFFFADPSC-SJOKHIZPCG-UHFFFADPSC-NUHFF-NUHFF-NUHFF-ZZZ

Original Data for the target compound can be accessed on the repository Chemotion:

**Type**	**DOI**	**Owner**[1]
**6b**	SJOKHIZPCGZHAT-UHFFFAOYSA-N	
1H NMR	https://dx.doi.org/10.14272/SJOKHIZPCGZHAT-UHFFFAOYSA-N/1HNMR	A
13C NMR	https://dx.doi.org/10.14272/SJOKHIZPCGZHAT-UHFFFAOYSA-N/13CNMR	A
Mass	https://dx.doi.org/10.14272/SJOKHIZPCGZHAT-UHFFFAOYSA-N/Mass	A
HRMS	https://dx.doi.org/10.14272/SJOKHIZPCGZHAT-UHFFFAOYSA-N/Mass.1	A
IR	https://dx.doi.org/10.14272/SJOKHIZPCGZHAT-UHFFFAOYSA-N/IR	A

[1] A = data of the authors; B = data provided by others

5.5 1,3-Bis((3,5-dichlorophenyl)thio)bicyclo[1.1.1]pentane (**6c**)

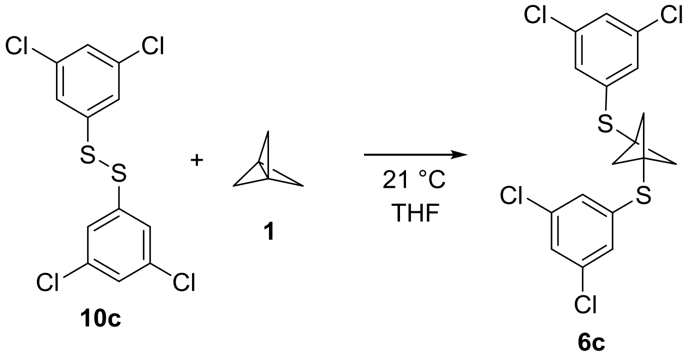

Name {P1|**6c**}: 1,3-Bis((3,5-dichlorophenyl)thio)bicyclo[1.1.1]pentane; Formula: $C_{17}H_{12}Cl_4S_2$; Molecular Mass: 422.2192; Exact Mass: 419.9135;
Smiles: Clc1cc(SC23CC(C2)(C3)Sc2cc(Cl)cc(c2)Cl)cc(c1)Cl;
InChIKey: DHAJMGHBXKCZPF-UHFFFAOYSA-N

According to General Procedure a: {A|**10c**} Bis(3,5-dichlorophenyl) disulfide (0.513 g, 1.44 mmol, 3.00 equiv); {B|**1**} [1.1.1]Propellane (0.0318 g, 0.481 mmol, 1.00 equiv); {S1} Tetrahydrofuran (1.0 mL); Yield {P1|**6c**} = 96% (0.194 g, 0.459 mmol).
The obtained crude product was purified *via* flash-chromatography on silica gel using pentane. The product was obtained as a white solid. m.p. 95 °C. R_f = 0.72 (pentane). ^{1}H NMR (400 MHz, $CDCl_3$, ppm) δ = 7.30–7.28 (m, 6H, Ar-H), 2.12 (s, 6H, 3xCH_2); ^{13}C NMR (100 MHz, $CDCl_3$, ppm) δ = 136.6 (C_{quat}, 2xC_{Ar}S), 135.2 (C_{quat}, 4xC_{Ar}Cl), 131.3 (+, 4xCH_{Ar}), 128.4 (+, 2xCH_{Ar}), 57.8 (+, 3xCH_2), 42.5 (C_{quat}, 2xCCH_2); EI (***m/z***, 70 eV, 100 °C): 424/422/420 (0.6/1/0.5) $[M]^+$, 247/245/243 (13/64/100) $[M–C_6H_3Cl_2S]^+$, 210/208 (13/18) $[M–C_6H_3Cl_2S–Cl]^+$, 179/177 (4/7) $[C_6H_3Cl_2S]^+$, 144/142 (3/8) $[C_6H_3ClS]^+$; HRMS (EI, 70 eV): Calcd for $C_{17}H_{12}{}^{35}Cl_4{}^{32}S_2$ $[M]^+$: 419.9134, found 419.9135; IR (ATR, ṽ) = 3118, 3081, 3054, 3000, 2963, 2915, 2876, 2850, 1742, 1568, 1553, 1503, 1445, 1421, 1401, 1378, 1360, 1288, 1203, 1137, 1125, 1096, 1050, 1018, 990, 926, 894, 874, 837, 790, 660, 561, 530, 521, 507, 463, 428, 409, 390 cm^{-1}.

The conditions for this reaction were deposited at Chemotion-repository:

https://dx.doi.org/10.14272/reaction/SA-FUHFF-UHFFFADPSC-DHAJMGHBXK-UHFFFADPSC-NUHFF-NUHFF-NUHFF-ZZZ

Original Data for the target compound can be accessed on the repository Chemotion:

**Type**	**DOI**	**Owner**[1]
**6c**	DHAJMGHBXKCZPF-UHFFFAOYSA-N	
1H NMR	https://dx.doi.org/10.14272/DHAJMGHBXKCZPF-UHFFFAOYSA-N/1HNMR	A
13C NMR	https://dx.doi.org/10.14272/DHAJMGHBXKCZPF-UHFFFAOYSA-N/13CNMR	A
Mass	https://dx.doi.org/10.14272/DHAJMGHBXKCZPF-UHFFFAOYSA-N/Mass	A
HRMS	https://dx.doi.org/10.14272/DHAJMGHBXKCZPF-UHFFFAOYSA-N/Mass.1	A
IR	https://dx.doi.org/10.14272/DHAJMGHBXKCZPF-UHFFFAOYSA-N/IR	A

[1] A = data of the authors; B = data provided by others

5.6 1,3-Bis(*p*-tolylthio)bicyclo[1.1.1]pentane (**6d**)

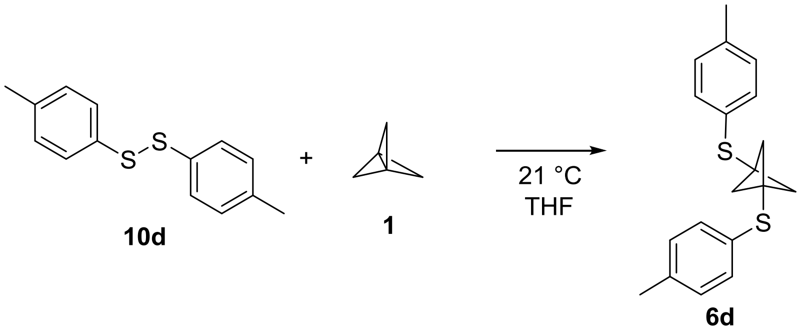

Name {P1|**6d**}: 1,3-Bis(*p*-tolylthio)bicyclo[1.1.1]pentane; Formula: $C_{19}H_{20}S_2$; Molecular Mass: 312.4921; Exact Mass: 312.1006;
Smiles: Cc1ccc(cc1)SC12CC(C1)(C2)Sc1ccc(cc1)C;
InChIKey: NZNGNSDNZKZZOJ-UHFFFAOYSA-N

According to General Procedure a: {A|**10d**} Bis(4-methylphenyl) disulfide (0.386 g, 1.57 mmol, 3.00 equiv); {B|**1**} [1.1.1]Propellane (0.0345 g, 0.522 mmol, 1.00 equiv); {S1} Tetrahydrofuran (1.0 mL); Yield {P1|**6d**} = 96% (0.156 g, 0.499 mmol).
The obtained crude product was purified *via* flash-chromatography on silica gel using pentane to pentane/diethyl ether 50:1. The product was obtained as a white solid. m.p. 50 °C. R_f = 0.51 (pentane/diethyl ether 50:1).
^{1}H NMR (400 MHz, $CDCl_3$, ppm) δ = 7.29–7.27 (m, 4H, Ar-H), 7.10 (d, *J* = 7.9 Hz, 4H, Ar-H), 2.33 (s, 6H, 2xCH_3), 1.97 (s, 6H, 3xCH_2); ^{13}C NMR (100 MHz, $CDCl_3$, ppm) δ = 138.2 (C_{quat}, 2x$C_{Ar}CH_3$), 134.2 (+, 4xCH_{Ar}), 129.8 (+, 4xCH_{Ar}), 129.6 (C_{quat}, 2x$C_{Ar}S$), 57.3 (–, 3xCH_2), 42.7 (C_{quat}, 2xCCH_2), 21.3 (+, 2xCH_3); EI (***m/z***, 70 eV, 60 °C): 312 (8) $[M]^+$, 189 (100) $[M–C_7H_7S]^+$, 123 (45) $[C_7H_7S]^+$, 91 (18) $[C_7H_7]^+$; HRMS (EI, 70 eV): Calcd for $C_{19}H_{20}{}^{32}S_2$ $[M]^+$: 312.1007, found 312.1006; IR (ATR, ṽ) = 2989, 2966, 2912, 2871, 1900, 1642, 1596, 1565, 1490, 1445, 1397, 1377, 1299, 1197, 1179, 1147, 1132, 1118, 1102, 1094, 1040, 1017, 929, 892, 809, 707, 633, 554, 513, 483, 415, 394 cm^{-1}.

The conditions for this reaction were deposited at Chemotion-repository:
https://dx.doi.org/10.14272/reaction/SA-FUHFF-UHFFFADPSC-NZNGNSDNZK-UHFFFADPSC-NUHFF-NUHFF-NUHFF-ZZZ

Original Data for the target compound can be accessed on the repository Chemotion:

**Type**	**DOI**	**Owner**[1]
**6d**	NZNGNSDNZKZZOJ-UHFFFAOYSA-N	
1H NMR	https://dx.doi.org/10.14272/NZNGNSDNZKZZOJ-UHFFFAOYSA-N/1HNMR	A
13C NMR	https://dx.doi.org/10.14272/NZNGNSDNZKZZOJ-UHFFFAOYSA-N/13CNMR	A
Mass	https://dx.doi.org/10.14272/NZNGNSDNZKZZOJ-UHFFFAOYSA-N/Mass	A
HRMS	https://dx.doi.org/10.14272/NZNGNSDNZKZZOJ-UHFFFAOYSA-N/Mass.1	A
IR	https://dx.doi.org/10.14272/NZNGNSDNZKZZOJ-UHFFFAOYSA-N/IR	A

[1] A = data of the authors; B = data provided by others

5.7 1,3-Bis((4-methoxyphenyl)thio)bicyclo[1.1.1]pentane (**6e**)

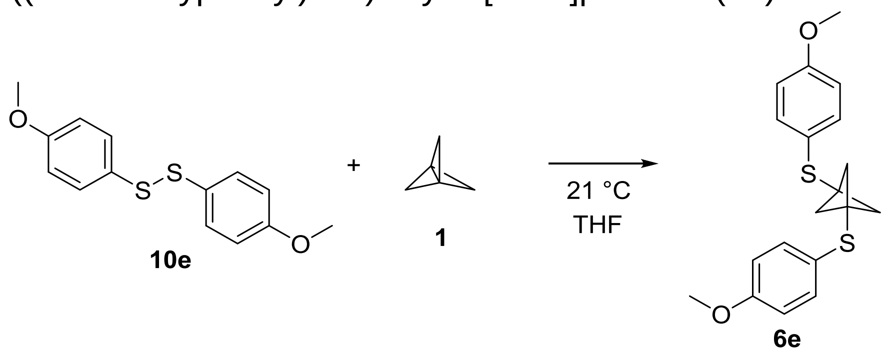

Name {P1|**6e**}: 1,3-Bis((4-methoxyphenyl)thio)bicyclo[1.1.1]pentane; Formula: $C_{19}H_{20}O_2S_2$; Molecular Mass: 344.4909; Exact Mass: 344.0905;
Smiles: COc1ccc(cc1)SC12CC(C1)(C2)Sc1ccc(cc1)OC
InChIKey: AWYXHOMVLAPYSJ-UHFFFAOYSA-N

According to General Procedure a: {A|**10e**} Bis(4-methoxyphenyl) disulfide (0.436 g, 1.57 mmol, 3.00 equiv); {B|**1**} [1.1.1]Propellane (0.0345 g, 0.522 mmol, 1.00 equiv); {S1} Tetrahydrofuran (1.0 mL); Yield {P1|**6e**} = 94% (0.169 g, 0.491 mmol).
The obtained crude product was purified *via* flash-chromatography on silica gel using pentane/diethyl ether 100:1. The product was obtained as a white solid. m.p. 66 °C. R_f = 0.24 (pentane/diethyl ether 100:1).
^{1}H NMR (400 MHz, $CDCl_3$, ppm) δ = 7.34–7.30 (m, 4H, Ar-H), 6.84–6.80 (m, 4H, Ar-H), 3.79 (s, 6H, 2xCH_3), 1.89 (s, 6H, 3xCH_2); ^{13}C NMR (100 MHz, $CDCl_3$, ppm) δ = 159.9 (C_{quat}, 2xC_{Ar}O), 136.2 (+, 4xCH_{Ar}), 123.7 (C_{quat}, 2xC_{Ar}S), 114.6 (+, 4xCH_{Ar}), 56.9 (–, 3xCH_2), 55.4 (+, 2xCH_3), 43.0 (C_{quat}, 2xCCH_2); EI (***m/z***, 70 eV, 70 °C): 344 (18) $[M]^+$, 205 (100) $[M–C_7H_7OS]^+$, 139 (69) $[C_7H_7OS]^+$; HRMS (EI, 70 eV): Calcd for $C_{19}H_{20}O_2{}^{32}S_2$ $[M]^+$: 344.0906, found 344.0905; IR (ATR, ṽ) = 2983, 2958, 2919, 2850, 2836, 1737, 1589, 1570, 1490, 1462, 1441, 1404, 1377, 1285, 1239, 1198, 1183, 1173, 1132, 1098, 1057, 1030, 1007, 922, 891, 875, 829, 813, 798, 758, 714, 663, 640, 628, 584, 555, 545, 524, 503, 487, 446, 405, 387, 381 cm^{-1}.

The conditions for this reaction were deposited at Chemotion-repository:

https://dx.doi.org/10.14272/reaction/SA-FUHFF-UHFFFADPSC-AWYXHOMVLA-UHFFFADPSC-NUHFF-NUHFF-NUHFF-ZZZ

Original Data for the target compound can be accessed on the repository Chemotion:

**Type**	**DOI**	**Owner**[1]
**6e**	AWYXHOMVLAPYSJ-UHFFFAOYSA-N	
1H NMR	https://dx.doi.org/10.14272/AWYXHOMVLAPYSJ-UHFFFAOYSA-N/1HNMR	A
13C NMR	https://dx.doi.org/10.14272/AWYXHOMVLAPYSJ-UHFFFAOYSA-N/13CNMR	A
Mass	https://dx.doi.org/10.14272/AWYXHOMVLAPYSJ-UHFFFAOYSA-N/Mass	A
HRMS	https://dx.doi.org/10.14272/AWYXHOMVLAPYSJ-UHFFFAOYSA-N/Mass.1	A
IR	https://dx.doi.org/10.14272/AWYXHOMVLAPYSJ-UHFFFAOYSA-N/IR	A

[1] A = data of the authors; B = data provided by others

5.8 1-Iodo-2-phenylbenzene (**17**)

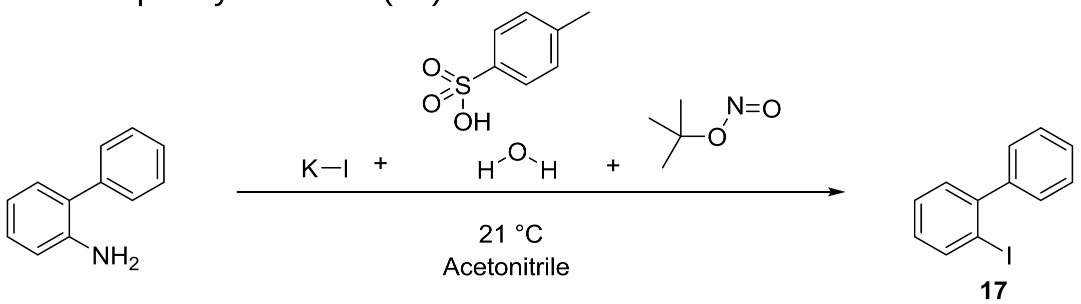

17

Name (**17**): 1-Iodo-2-phenylbenzene; Formula: $C_{12}H_9I$; Molecular Mass: 280.1043; Exact Mass: 279.9749;
Smiles: Ic1ccccc1c1ccccc1; InChIKey: QFUYDAGNUJWBSM-UHFFFAOYSA-N

Tert-butyl nitrite (1.83 g, 2.11 mL, 18.0 mmol, 3.00 equiv) was added to a solution of *para*-toluenesulfonic acid monohydrate (3.37 g, 18.0 mmol, 3.00 equiv) in acetonitrile (25.0 mL), followed by 2-phenylaniline (1.00 g, 5.90 mmol, 1.00 equiv). After consumption of the starting material, potassium iodide (4.90 g, 30.0 mmol, 5.00 equiv), dissolved in 5 mL water, was added slowly and the mixture was stirred for 2 h at rt. The product was extracted with ethyl acetate and washed with sat. $Na_2S_2O_3$ and water. The aqueous phase was back-extracted with ethyl acetate. The combined organic layers were dried by the addition of Na_2SO_4. The mixture was filtered through a glass funnel and the solvent was evaporated under reduced pressure.
The obtained crude product was purified via flash-chromatography on silica gel using cyclohexane giving the target compound **17** as a yellow oil in 83% yield (1.38 g, 4.93 mmol). R_f = 0.51 (cyclohexane).
^{1}H NMR (400 MHz, $CDCl_3$, ppm) δ = 7.97 (dd, *J* = 7.9, 1.2 Hz, 1H, Ar-H), 7.48–7.37 (m, 4H, Ar-H), 7.36–7.30 (m, 3H, Ar-H), 7.04 (td, *J* = 7.6, 1.9 Hz, 1H, Ar-H); ^{13}C NMR (100 MHz, $CDCl_3$, ppm) δ = 146.8 (C_{quat}, $C_{Ar}C_{Ar}$), 144.4 (C_{quat}, $C_{Ar}C_{Ar}$), 139.6 (+, CH_{Ar}), 130.2 (+, CH_{Ar}), 129.4 (+, 2xCH_{Ar}), 128.9 (+, CH_{Ar}), 128.2 (+, CH_{Ar}), 128.1 (+, 2xCH_{Ar}), 127.8 (+, CH_{Ar}), 98.8 (C_{quat}, C_{Ar}I). In accordance with literature: D. Chen, G. Shi, H. Jiang, Y. Zhang, Y. Zhang, *Org. Lett.*, **2016**, *18*, 2130–2133.

The conditions for this reaction were deposited at Chemotion-repository:

https://dx.doi.org/10.14272/reaction/SA-FUHFF-UHFFFADPSC-QFUYDAGNUJ-UHFFFADPSC-NUHFF-NUHFF-NUHFF-ZZZ

Original Data for the target compound can be accessed on the repository Chemotion:

**Type**	**DOI**	**Owner**[1]
**17**	QFUYDAGNUJWBSM-UHFFFAOYSA-N	
1H NMR	https://dx.doi.org/10.14272/QFUYDAGNUJWBSM-UHFFFAOYSA-N/1HNMR	A
13C NMR	https://dx.doi.org/10.14272/QFUYDAGNUJWBSM-UHFFFAOYSA-N/13CNMR	A

[1] A = data of the authors; B = data provided by others

5.9 1-Phenyl-2-[(2-phenylphenyl)disulfanyl]benzene (**10f**)

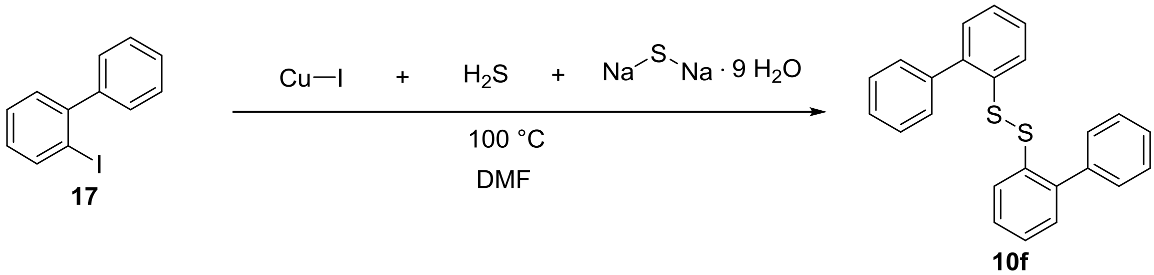

Name (**10f**): 1-Phenyl-2-[(2-phenylphenyl)disulfanyl]benzene; Formula: $C_{24}H_{18}S_2$; Molecular Mass: 370.5297; Exact Mass: 370.0850;
Smiles: c1ccc(cc1)c1ccccc1SSc1ccccc1c1ccccc1;
InChIKey: JKPTZWVDCSARTM-UHFFFAOYSA-N

1-Iodo-2-phenylbenzene (1.27 g, 4.50 mmol, 1.00 equiv), copper(I) iodide (86.4 mg, 453 µmol, 0.100 equiv), sulfur (145 mg, 4.50 mmol, 1.00 equiv) and sodium sulfide nonahydrate (1.09 g, 4.50 mmol, 1.00 equiv) were mixed in a vial under argon atmosphere. N,N-dimethylformamide (8.50 mL) was added and the reaction mixture was heated at 100 °C for 16 h. The reaction was quenched by the addition of 1 mL H_2O. The aqueous layer was extracted with ethyl acetate. The combined organic phase was dried by the addition of Na_2SO_4, filtered and the solvent was removed under reduced pressure.
The obtained crude product was purified via flash-chromatography on silica gel using cyclohexane. After trituration with diethyl ether, the target compound **10f** was obtained in 39% yield (0.658 g, 1.78 mmol) as a white solid. R_f = 0.22 (cyclohexane).

^{1}H NMR (400 MHz, $CDCl_3$, ppm) δ = 7.60 (dd, J = 6.7, 2.2 Hz, 2H, Ar-H), 7.47–7.37 (m, 10H, Ar-H), 7.28–7.20 (m, 6H, Ar-H).; ^{13}C NMR (100 MHz, $CDCl_3$, ppm) δ = 141.4 (C_{quat}, 2xC_{Ar}), 139.8 (C_{quat}, 2xC_{Ar}), 135.1 (C_{quat}, 2xC_{Ar}), 130.2 (+, 2xCH_{Ar}), 129.7 (+, 4xCH_{Ar}), 128.4 (+, 2xCH_{Ar}), 128.4 (+, 4xCH_{Ar}), 127.9 (+, 2xCH_{Ar}), 127.3 (+, 2xCH_{Ar}), 126.7 (+, 2xCH_{Ar}). In accordance with literature: K. Nishino, Y. Ogiwara, N. Sakai, *Eur. J. Org. Chem.*, **2017**, *2017*, 5892–5895.

The conditions for this reaction were deposited at Chemotion-repository:

https://dx.doi.org/10.14272/reaction/SA-FUHFF-UHFFFADPSC-JKPTZWVDCS-UHFFFADPSC-NUHFF-NUHFF-NUHFF-ZZZ

Original Data for the target compound can be accessed on the repository Chemotion:

**Type**	**DOI**	**Owner**[1]
**10f**	JKPTZWVDCSARTM-UHFFFAOYSA-N	
1H NMR	https://dx.doi.org/10.14272/JKPTZWVDCSARTM-UHFFFAOYSA-N/1HNMR	A
13C NMR	https://dx.doi.org/10.14272/JKPTZWVDCSARTM-UHFFFAOYSA-N/13CNMR	A

[1] A = data of the authors; B = data provided by others

5.10 1,3-Bis([1,1'-biphenyl]-2-ylthio)bicyclo[1.1.1]pentane (**6f**)

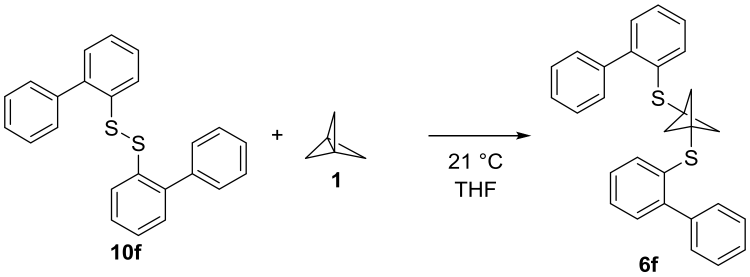

Name {P1|**6f**}: 1,3-Bis([1,1'-biphenyl]-2-ylthio)bicyclo[1.1.1]pentane; Formula: $C_{29}H_{24}S_2$; Molecular Mass: 436.6309; Exact Mass: 436.1319;
Smiles: c1ccc(cc1)c1ccccc1SC12CC(C1)(C2)Sc1ccccc1c1ccccc1;
InChIKey: PINRSYZIAIUFQE-UHFFFAOYSA-N

According to General Procedure a: {A|**10f**} 1-Phenyl-2-[(2-phenylphenyl)disulfanyl]benzene (0.117 g, 0.316 mmol, 3.00 equiv); {B|**1**} [1.1.1]Propellane (0.00697 g, 0.105 mmol, 1.00 equiv); {S1} Tetrahydrofuran (1.0 mL); Yield {P1|**6f**} = 61% (0.0280 g, 0.0641 mmol).
The obtained crude product was purified via flash-chromatography on silica gel using pentane to pentane/diethyl ether 200:1. The product was obtained as a white solid. m.p. 109 °C. R_f = 0.27 (pentane/diethyl ether 200:1).
^{1}H NMR (400 MHz, $CDCl_3$, ppm) δ = 7.49 (dt, *J* = 7.2, 1.2 Hz, 2H, Ar-H), 7.39–7.27 (m, 15H, Ar-H), 7.26 –7.22 (m, 1H, Ar-H), 1.78 (s, 6H, 3xCH_2); ^{13}C NMR (100 MHz, $CDCl_3$, ppm) δ = 145.1 (C_{quat}, 2xC_{Ar}), 141.1 (C_{quat}, 2xC_{Ar}), 134.5 (+, 2xCH_{Ar}), 132.2 (C_{quat}, 2xC_{Ar}S), 130.7 (+, 2xCH_{Ar}), 129.9 (+, 4xCH_{Ar}), 127.8 (+, 2xCH_{Ar}), 127.8 (+, 4xCH_{Ar}), 127.7 (+, 2xCH_{Ar}), 127.4 (+, 2xCH_{Ar}), 57.6 (–, 3xCH_2), 42.6 (C_{quat}, 2xCCH_2); EI (***m/z***, 70 eV, 160 °C): 436 (2) $[M]^+$, 251 (100) $[M–C_{12}H_9S]^+$, 185 (55) $[C_{12}H_9S]^+$, 184 (67) $[C_{12}H_9S–H]^+$; HRMS (EI, 70 eV): Calcd for $C_{29}H_{24}{}^{32}S_2$ [M]+: 436.1321, found 436.1319; IR (ATR, ṽ) = 3053, 2993, 2962, 2910, 2870, 1584, 1494, 1459, 1443, 1424, 1198, 1157, 1123, 1069, 1038, 1007, 925, 870, 769, 751, 700, 680, 613, 552, 535, 501, 467, 435, 411, 380 cm^{-1}.

The conditions for this reaction were deposited at Chemotion-repository:

https://dx.doi.org/10.14272/reaction/SA-FUHFF-UHFFFADPSC-PINRSYZIAI-UHFFFADPSC-NUHFF-NUHFF-NUHFF-ZZZ

Original Data for the target compound can be accessed on the repository Chemotion:

**Type**	**DOI**	**Owner**[1]
**6f**	PINRSYZIAIUFQE-UHFFFAOYSA-N	
1H NMR	https://dx.doi.org/10.14272/PINRSYZIAIUFQE-UHFFFAOYSA-N/1HNMR	A
13C NMR	https://dx.doi.org/10.14272/PINRSYZIAIUFQE-UHFFFAOYSA-N/13CNMR	A
Mass	https://dx.doi.org/10.14272/PINRSYZIAIUFQE-UHFFFAOYSA-N/Mass	A
HRMS	https://dx.doi.org/10.14272/PINRSYZIAIUFQE-UHFFFAOYSA-N/Mass.1	A
IR	https://dx.doi.org/10.14272/PINRSYZIAIUFQE-UHFFFAOYSA-N/IR	A

[1] A = data of the authors; B = data provided by others

5.11 1,3-Bis(phenylthio)bicyclo[1.1.1]pentane (**6a**), 3,3'-Bis(phenylthio)-1,1'-bi(bicyclo[1.1.1]pentane) (**11a**)

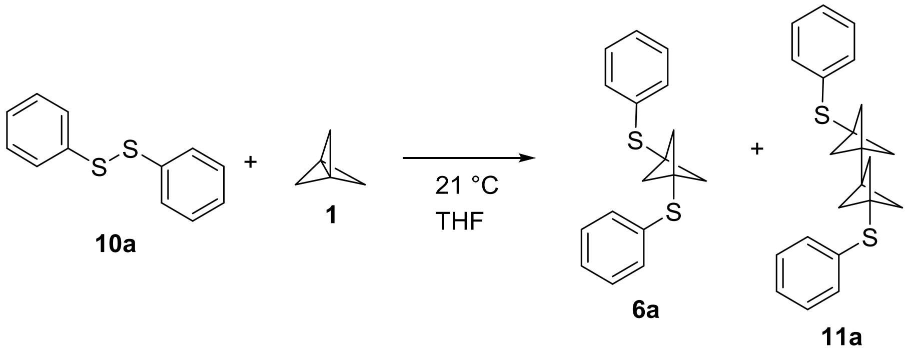

Name {P1|**6a**}: 1,3-Bis(phenylthio)bicyclo[1.1.1]pentane; Formula: $C_{17}H_{16}S_2$; Molecular Mass: 284.4389; Exact Mass: 284.0693;
Smiles: c1ccc(cc1)SC12CC(C1)(C2)Sc1ccccc1;
InChIKey: GHXRODNNSAZUSG-UHFFFAOYSA-N

Name {P2|**11a**}: 3,3'-Bis(phenylthio)-1,1'-bi(bicyclo[1.1.1]pentane); Formula: $C_{22}H_{22}S_2$; Molecular Mass: 350.5401; Exact Mass: 350.1163;
Smiles: c1ccc(cc1)SC12CC(C1)(C2)C12CC(C1)(C2)Sc1ccccc1;
InChIKey: UFJZAHXFSRPBMA-UHFFFAOYSA-N

According to General Procedure b: {A|**10a**} Diphenyl disulfide (43.7 mg, 0.200 mmol, 1.00 equiv); {B|**1**} [1.1.1]Propellane (26.4 mg, 0.400 mmol, 2.00 equiv); {S1} Tetrahydrofuran (1.0 mL); Yield {P1|**6a**} = 32% (18.0 mg, 0.0633 mmol); {P2|**11a**} = 10% (7.0 mg, 0.0200 mmol).
The obtained crude product was purified *via* preparative TLC using pentane. Two fractions were obtained: Product {P1|**6a**}: 18 mg (32%) white solid, m.p. 54 °C. Product {P2|**11a**}: 7.0 mg (10%) white solid, m.p. 134 °C; R_f {P2|**11a**} = 0.10 (pentane).

Analysis of Product {P2|**11a**}:
^{1}H NMR (400 MHz, $CDCl_3$, ppm) δ = 7.42–7.39 (m, 4H, Ar-H), 7.31–7.27 (m, 6H, Ar-H), 1.73 (s, 12H, 3xCH_2); ^{13}C NMR (100 MHz, $CDCl_3$, ppm) δ = 134.0 (C_{quat}, 2xC_{Ar}S), 133.8 (+, 4xCH_{Ar}), 128.9 (+, 4xCH_{Ar}), 127.7 (+, 2xCH_{Ar}), 53.3 (–, 6xCH_2), 41.6 (C_{quat}, 2xCCH_2), 40.3 (C_{quat}, 2xCCH_2); EI (***m/z***, 70 eV, 100 °C): 350 (4) $[M]^+$, 241 (32) $[M–C_6H_5S]^+$, 131 (100) $[M–(C_6H_5S)_2–H]^+$, 109 (15) $[C_6H_5S]^+$, 91 (53) $[M–C_6H_5S–C_9H_{10}S]^+$; HRMS (EI, 70 eV): Calcd for $C_{22}H_{22}{}^{32}S_2$ [M]+: 350.1161, found 350.1163; IR (ATR, ṽ) = 2979, 2959, 2921, 2905, 2868, 1737, 1578, 1472, 1438, 1391, 1302, 1261, 1203, 1177, 1142, 1130, 1086, 1067, 1020, 999, 909, 887, 860, 802, 779, 744, 704, 688, 660, 615, 582, 550, 506, 477, 446, 421, 390 cm^{-1}.

The conditions for this reaction were deposited at Chemotion-repository:
https://dx.doi.org/10.14272/reaction/SA-FUHFF-UHFFFADPSC-HQLMMCQLNP-UHFFFADPSC-NUHFF-NUHFF-NUHFF-ZZZ

Original Data for the target compound can be accessed on the repository Chemotion:

Type	DOI	Owner[1]
**6a**	GHXRODNNSAZUSG-UHFFFAOYSA-N	
1H NMR	https://dx.doi.org/10.14272/GHXRODNNSAZUSG-UHFFFAOYSA-N/1HNMR.1	A

**11a**	UFJZAHXFSRPBMA-UHFFFAOYSA-N	
1H NMR	https://dx.doi.org/10.14272/UFJZAHXFSRPBMA-UHFFFAOYSA-N/1HNMR	A
13C NMR	https://dx.doi.org/10.14272/UFJZAHXFSRPBMA-UHFFFAOYSA-N/13CNMR	A
Mass	https://dx.doi.org/10.14272/UFJZAHXFSRPBMA-UHFFFAOYSA-N/Mass	A
HRMS	https://dx.doi.org/10.14272/UFJZAHXFSRPBMA-UHFFFAOYSA-N/Mass.1	A
IR	https://dx.doi.org/10.14272/UFJZAHXFSRPBMA-UHFFFAOYSA-N/IR	A

[1] A = data of the authors; B = data provided by others

5.12 1,3-Bis((4-chlorophenyl)thio)bicyclo[1.1.1]pentane (**6b**), 3,3'-Bis((4-chlorophenyl)thio)-1,1'-bi(bicyclo[1.1.1]pentane) (**11b**)

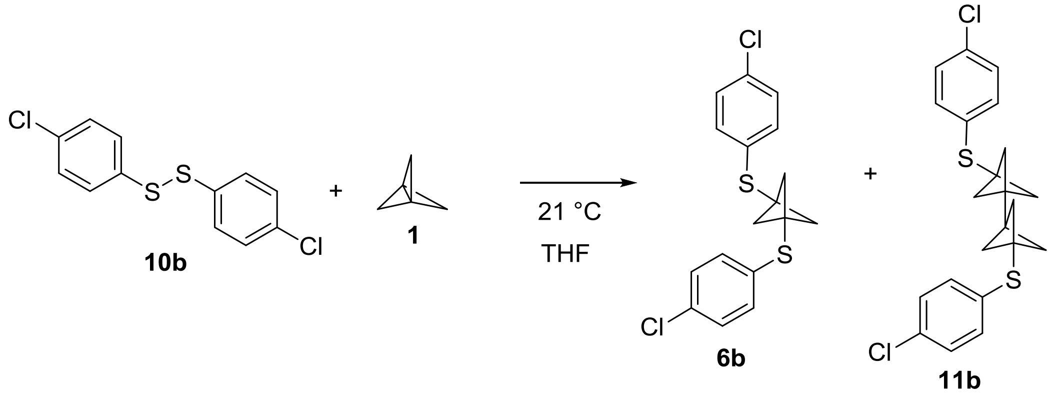

Name {P1|**6b**}: 1,3-Bis((4-chlorophenyl)thio)bicyclo[1.1.1]pentane; Formula: $C_{17}H_{14}Cl_2S_2$; Molecular Mass: 353.3291; Exact Mass: 351.9914;
Smiles: Clc1ccc(cc1)SC12CC(C1)(C2)Sc1ccc(cc1)Cl;
InChIKey: SJOKHIZPCGZHAT-UHFFFAOYSA-N

Name {P2|**11b**}: 3,3'-Bis((4-chlorophenyl)thio)-1,1'-bi(bicyclo[1.1.1]pentane); Formula: $C_{22}H_{20}Cl_2S_2$; Molecular Mass: 419.4302; Exact Mass: 418.0383;
Smiles: Clc1ccc(cc1)SC12CC(C1)(C2)C12CC(C1)(C2)Sc1ccc(cc1)Cl;
InChIKey: PDIYSPWIMSKCME-UHFFFAOYSA-N

According to General Procedure b: {A|**10b**} 1-chloro-4-[(4-chlorophenyl)disulfanyl]benzene (0.0574 g, 0.200 mmol, 1.00 equiv); {B|**1**} [1.1.1]Propellane (0.0264 g, 0.400 mmol, 2.00 equiv); {S1} Tetrahydrofuran (1.0 mL); Yield {P1|**6b**} = 34% (0.0240 g, 0.0679 mmol); {P2|**11b**} = 15% (0.0130 g, 0.0310 mmol).
The obtained crude product was purified *via* preparative TLC using pentane. Two fractions were obtained: Product {P1|**6b**}: 24 mg (34%) white solid, m.p. 94 °C. Product {P2|**11b**}: 13 mg (15%) white solid, m.p. 155 °C; R_f {P2|**11b**} = 0.30 (pentane).

Analysis of Product {P2|**11b**}:
^{1}H NMR (400 MHz, $CDCl_3$, ppm) δ = 7.35–7.24 (m, 8H, Ar-H), 1.72 (s, 12H, 6xCH_2); ^{13}C NMR (100 MHz, $CDCl_3$, ppm) δ = 135.1 (+, 4xCH$_{Ar}$), 134.0 (C_{quat}, 2xC_{Ar}S), 132.4 (C_{quat}, 2xC_{Ar}Cl), 129.1 (+, 4xCH$_{Ar}$), 53.3 (–, 6xCH_2), 41.6 (C_{quat}, 2xCCH$_2$), 40.3 (C_{quat}, 2xCCH$_2$); EI (***m/z***, 70 eV, 110 °C): 422/420/418 (1/2/3) $[M]^+$, 275 (14) $[M–C_6H_4ClS]^+$, 143 (19) $[C_6H_4ClS]^+$, 131 (100) $[M–(C_6H_4ClS)_2–H]^+$, 91 (77) $[M–C_6H_4ClS–C_9H_9ClS]^+$; HRMS (EI, 70 eV): Calcd for $C_{22}H_{20}{}^{35}Cl_2{}^{32}S_2$ [M]+: 418.0382, found 418.0383; IR

(ATR, ṽ) = 2982, 2959, 2919, 2904, 2868, 2851, 1907, 1735, 1655, 1571, 1472, 1441, 1385, 1259, 1207, 1201, 1173, 1142, 1116, 1091, 1011, 907, 887, 860, 822, 800, 745, 704, 557, 543, 506, 446, 390 cm^{-1}.

The conditions for this reaction were deposited at Chemotion-repository: https://dx.doi.org/10.14272/reaction/SA-FUHFF-UHFFFADPSC-NNXNECGMTY-UHFFFADPSC-NUHFF-NUHFF-NUHFF-ZZZ

Original Data for the target compound can be accessed on the repository Chemotion:

Type	DOI	Owner[1]
**6b**	SJOKHIZPCGZHAT-UHFFFAOYSA-N	
1H NMR	https://dx.doi.org/10.14272/SJOKHIZPCGZHAT-UHFFFAOYSA-N/1HNMR.1	A
**11b**	PDIYSPWIMSKCME-UHFFFAOYSA-N	
1H NMR	https://dx.doi.org/10.14272/PDIYSPWIMSKCME-UHFFFAOYSA-N/1HNMR	A
13C NMR	https://dx.doi.org/10.14272/PDIYSPWIMSKCME-UHFFFAOYSA-N/13CNMR	A
Mass	https://dx.doi.org/10.14272/PDIYSPWIMSKCME-UHFFFAOYSA-N/Mass	A
HRMS	https://dx.doi.org/10.14272/PDIYSPWIMSKCME-UHFFFAOYSA-N/Mass.1	A
IR	https://dx.doi.org/10.14272/PDIYSPWIMSKCME-UHFFFAOYSA-N/IR	A

[1] A = data of the authors; B = data provided by others

5.13 1,3-Bis((3,5-dichlorophenyl)thio)bicyclo[1.1.1]pentane (**6c**), 3,3'-Bis((3,5-dichlorophenyl)thio)-1,1'-bi(bicyclo[1.1.1]pentane) (**11c**)

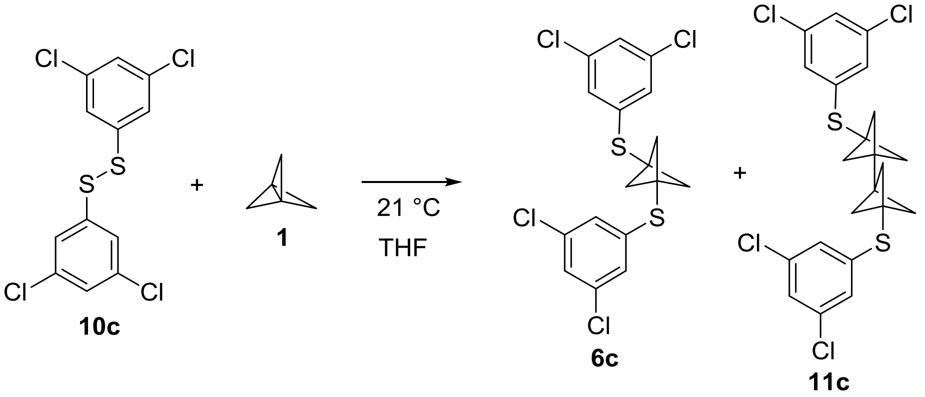

Name {P1|**6c**}: 1,3-Bis((3,5-dichlorophenyl)thio)bicyclo[1.1.1]pentane; Formula: $C_{17}H_{12}Cl_4S_2$; Molecular Mass: 422.2192; Exact Mass: 419.9135;
Smiles: Clc1cc(SC23CC(C2)(C3)Sc2cc(Cl)cc(c2)Cl)cc(c1)Cl;
InChIKey: DHAJMGHBXKCZPF-UHFFFAOYSA-N

Name {P2|**11c**}: 3,3'-Bis((3,5-dichlorophenyl)thio)-1,1'-bi(bicyclo[1.1.1]pentane); Formula: $C_{22}H_{18}Cl_4S_2$; Molecular Mass: 488.3203; Exact Mass: 485.9604;
Smiles: Clc1cc(SC23CC(C2)(C3)C23CC(C2)(C3)Sc2cc(Cl)cc(c2)Cl)cc(c1)Cl;
InChIKey: KGJVMZAWJCTKCI-UHFFFAOYSA-N

According to General Procedure b: {A|**10c**} 1,3-Dichloro-5-[(3,5-dichlorophenyl)disulfanyl]benzene (0.0712 g, 0.200 mmol, 1.00 equiv); {B|**1**} [1.1.1]Propellane (0.0264 g, 0.400 mmol, 2.00 equiv); {S1} Tetrahydrofuran (1.0 mL); Yield {P1|**6c**} = 34% (0.0290 g, 0.0687 mmol); {P2|**11c**} = 8% (0.00800 g, 0.0164 mmol).

The obtained crude product was purified *via* preparative TLC using pentane. Two fractions were obtained: Product {P1|**6c**}: 29 mg (34%) white solid, m.p. 95 °C. Product {P2|**11c**}: 8 mg (8%) white solid, m.p. 114 °C. R_f {P2|**11c**} = 0.49 (pentane).

Analysis of Product {P2|**11c**}:
^{1}H NMR (400 MHz, $CDCl_3$, ppm) δ = 7.27 (d, *J* = 1.8 Hz, 4H, Ar-H), 7.25 (t, *J* = 1.8 Hz, 2H, Ar-H), 1.82 (s, 12H, 6xCH_2); ^{13}C NMR (100 MHz, $CDCl_3$, ppm) δ = 137.7 (C_{quat}, 2xC_{Ar}S), 134.0 (C_{quat}, 4xC_{Ar}Cl), 130.9 (+, 4xCH_{Ar}), 127.7 (+, 2xCH_{Ar}), 53.6 (–, 6xCH_2), 41.3 (C_{quat}, 2xCCH_2), 40.6 (C_{quat}, 2xCCH_2); EI (***m/z***, 70 eV, 140 °C): 492/490/488/486 (1) $[M]^+$, 313/311/309 (2/8/11) $[M–C_6H_3Cl_2S]^+$, 131 (100) $[M–(C_6H_3Cl_2S)_2–H]^+$, 91 (50) $[M–C_6H_3Cl_2S–C_9H_8Cl_2S]^+$; HRMS (EI, 70 eV): Calcd for $C_{22}H_{18}{}^{35}Cl_4{}^{32}S_2$ [M]+: 485.9605, found 485.9604; IR (ATR, ṽ) = 381, 402, 428, 459, 496, 528, 545, 663, 734, 793, 837, 853, 863, 890, 990, 1006, 1023, 1052, 1095, 1129, 1140, 1177, 1211, 1261, 1288, 1364, 1378, 1401, 1418, 1445, 1463, 1551, 1605, 1689, 2853, 2874, 2908, 2921, 2959, 2986, 3072 cm^{-1}.

The conditions for this reaction were deposited at Chemotion-repository:
https://dx.doi.org/10.14272/reaction/SA-FUHFF-UHFFFADPSC-IPOJHZYBCZ-UHFFFADPSC-NUHFF-NUHFF-NUHFF-ZZZ

Original Data for the target compound can be accessed on the repository Chemotion:

**Type**	**DOI**	**Owner**[1]
**6c**	DHAJMGHBXKCZPF-UHFFFAOYSA-N	
1H NMR	https://dx.doi.org/10.14272/DHAJMGHBXKCZPF-UHFFFAOYSA-N/1HNMR.1	A
**11c**	KGJVMZAWJCTKCI-UHFFFAOYSA-N	
1H NMR	https://dx.doi.org/10.14272/KGJVMZAWJCTKCI-UHFFFAOYSA-N/1HNMR	A
13C NMR	https://dx.doi.org/10.14272/KGJVMZAWJCTKCI-UHFFFAOYSA-N/13CNMR	A
Mass	https://dx.doi.org/10.14272/KGJVMZAWJCTKCI-UHFFFAOYSA-N/Mass	A
HRMS	https://dx.doi.org/10.14272/KGJVMZAWJCTKCI-UHFFFAOYSA-N/Mass.1	A
IR	https://dx.doi.org/10.14272/KGJVMZAWJCTKCI-UHFFFAOYSA-N/IR	A

[1] A = data of the authors; B = data provided by others

5.14 1,3-Bis(*p*-tolylthio)bicyclo[1.1.1]pentane (**6d**), 3,3'-Bis(*p*-tolylthio)-1,1'-bi(bicyclo[1.1.1]pentane) (**11d**)

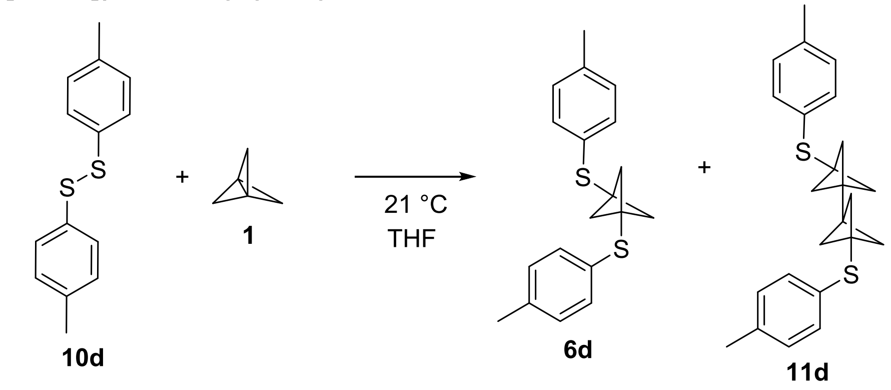

Name {P1|**6d**}: 1,3-Bis(*p*-tolylthio)bicyclo[1.1.1]pentane; Formula: $C_{19}H_{20}S_2$; Molecular Mass: 312.4921; Exact Mass: 312.1006;
Smiles: Cc1ccc(cc1)SC12CC(C1)(C2)Sc1ccc(cc1)C;
InChIKey: NZNGNSDNZKZZOJ-UHFFFAOYSA-N

Name {P2|**11d**}: 3,3'-Bis(*p*-tolylthio)-1,1'-bi(bicyclo[1.1.1]pentane); Formula: $C_{24}H_{26}S_2$; Molecular Mass: 378.5932; Exact Mass: 378.1476;
Smiles: Cc1ccc(cc1)SC12CC(C1)(C2)C12CC(C1)(C2)Sc1ccc(cc1)C;
InChIKey: AUAIDTHVAFAXGQ-UHFFFAOYSA-N

According to General Procedure b: {A|**10d**} 1-Methyl-4-[(4-methylphenyl)disulfanyl]benzene (0.0493 g, 0.200 mmol, 1.00 equiv); {B|**1**} [1.1.1]Propellane (0.0264 g, 0.400 mmol, 2.00 equiv); {S1} Tetrahydrofuran (1.0 mL); Yield {P1|**6d**} = 35% (0.0220 g, 0.0704 mmol); {P2|**11d**} = 12% (0.00900 g, 0.0238 mmol).
The obtained crude product was purified *via* preparative TLC using pentane. Two fractions were obtained: Product {P1|**6d**}: 22 mg (35%) white solid, m.p. 50 °C. Product {P2|**11d**}: 9 mg (12%) white solid, m.p. 143 °C; R_f {P2|**11d**} = 0.35 (pentane).

Analysis of Product {P2|**11d**}:
^{1}H NMR (400 MHz, $CDCl_3$, ppm) δ = 7.29 (d, J = 8.1 Hz, 4H, Ar-H), 7.09 (dd, J = 8.4, 0.8 Hz, 4H, Ar-H), 2.33 (s, 6H, 2xCH_3), 1.68 (s, 12H, 6xCH_2); ^{13}C NMR (100 MHz, $CDCl_3$, ppm) δ = 137.8 (C_{quat}, 2x$C_{Ar}CH_3$), 134.1 (+, 4xCH_{Ar}), 130.2 (C_{quat}, 2x$C_{Ar}S$), 129.7 (+, 4xCH_{Ar}), 53.2 (–, 6xCH_2), 41.7 (C_{quat}, 2xCCH_2), 40.1 (C_{quat}, 2xCCH_2), 21.3 (+, 2xCH_3); EI (***m/z***, 70 eV, 100 °C): 378 (4) $[M]^+$, 255 (21) $[M–C_7H_7S]^+$, 189 (29) $[M–C_{12}H_{13}S]^+$, 131 (100) $[M–(C_7H_7S)_2–H]^+$, 91 (76) $[M–C_7H_7S–C_{10}H_{12}S]^+$; HRMS (EI, 70 eV): Calcd for $C_{24}H_{26}{}^{32}S_2$ [M]+: 378.1477, found 378.1476; IR (ATR, ṽ) = 2979, 2961, 2919, 2905, 2868, 1735, 1487, 1459, 1441, 1397, 1377, 1299, 1261, 1203, 1177, 1142, 1128, 1102, 1094, 1038, 1017, 970, 942, 909, 888, 860, 809, 778, 734, 707, 635, 550, 507, 479, 445, 405 cm^{-1}.

The conditions for this reaction were deposited at Chemotion-repository:
https://dx.doi.org/10.14272/reaction/SA-FUHFF-UHFFFADPSC-RJKOTIRDIV-UHFFFADPSC-NUHFF-NUHFF-NUHFF-ZZZ

Original Data for the target compound can be accessed on the repository Chemotion:

**Type**	**DOI**	**Owner**[1]
**6d**	NZNGNSDNZKZZOJ-UHFFFAOYSA-N	
1H NMR	https://dx.doi.org/10.14272/NZNGNSDNZKZZOJ-UHFFFAOYSA-N/1HNMR.1	A
**11d**	AUAIDTHVAFAXGQ-UHFFFAOYSA-N	
1H NMR	https://dx.doi.org/10.14272/AUAIDTHVAFAXGQ-UHFFFAOYSA-N/1HNMR	A
13C NMR	https://dx.doi.org/10.14272/AUAIDTHVAFAXGQ-UHFFFAOYSA-N/13CNMR	A
Mass	https://dx.doi.org/10.14272/AUAIDTHVAFAXGQ-UHFFFAOYSA-N/Mass	A
HRMS	https://dx.doi.org/10.14272/AUAIDTHVAFAXGQ-UHFFFAOYSA-N/Mass.1	A
IR	https://dx.doi.org/10.14272/AUAIDTHVAFAXGQ-UHFFFAOYSA-N/IR	A

[1] A = data of the authors; B = data provided by others

5.15 1,3-Bis(benzylthio)bicyclo[1.1.1]pentane (**13**), 3,3'-Bis(benzylthio)-1,1'-bi(bicyclo[1.1.1]pentane) (**14**)

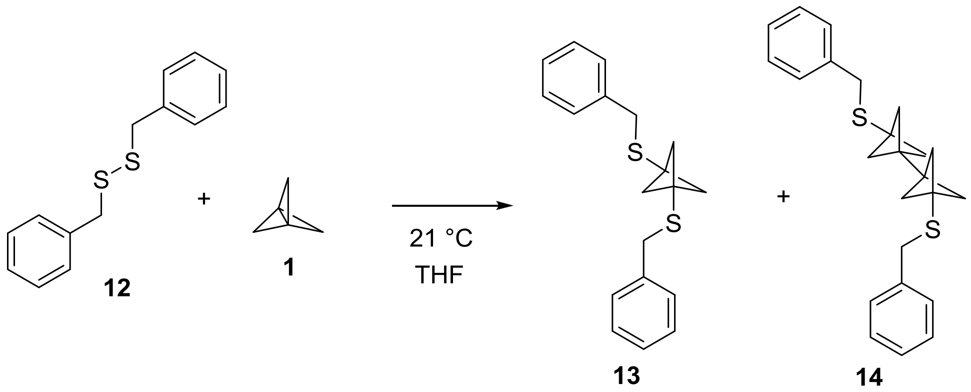

Name {P1|**13**}: 1,3-Bis(benzylthio)bicyclo[1.1.1]pentane; Formula: $C_{19}H_{20}S_2$; Molecular Mass: 312.4921; Exact Mass: 312.1006;
Smiles: c1ccc(cc1)CSC12CC(C1)(C2)SCc1ccccc1;
InChIKey: GXKYJSRHQMPAPZ-UHFFFAOYSA-N

Name {P2|**14**}: 3,3'-Bis(benzylthio)-1,1'-bi(bicyclo[1.1.1]pentane; Formula: $C_{24}H_{26}S_2$; Molecular Mass: 378.5932; Exact Mass: 378.1476;
Smiles: c1ccc(cc1)CSC12CC(C1)(C2)C12CC(C1)(C2)SCc1ccccc1;
InChIKey: IPSCQEYBZPKANH-UHFFFAOYSA-N

According to General Procedure a: {A|**12**} (Benzyldisulfanyl)methylbenzene (0.323 g, 1.31 mmol, 3.00 equiv); {B|**1**} [1.1.1]Propellane (0.0289 g, 0.437 mmol, 1.00 equiv); {S1} Tetrahydrofuran (1.0 mL); Yield {P1|**13**} = 40% (0.0540 g, 0.173 mmol); {P2|**14**} = traces.
The obtained crude product was purified via HPLC (C18, H_2O/ACN, 10:90 to 7:93). The product was obtained as a white solid. m.p. 64 °C. R_f {P2|**13**} = 0.10 (pentane).
Analysis of {P2|**13**}:
^{1}H NMR (400 MHz, $CDCl_3$, ppm) δ = 7.34–7.20 (m, 10H, Ar-H), 3.73 (s, 4H, 2xPhCH_2), 1.91 (s, 6H, 3xCCH_2); ^{13}C NMR (100 MHz, $CDCl_3$, ppm) δ = 138.4 (C_{quat}, 2x$C_{Ar}CH_2$), 128.9 (+, 4xCH_{Ar}), 128.6 (+, 4xCH_{Ar}), 127.2 (+, 2xCH_{Ar}), 57.0 (–, 3xCCH_2), 41.3 (C_{quat}, 2xCCH_2), 36.2 (–, 2xPhCH_2); EI (***m/z***, 70 eV, 80 °C): 221 (34) $[M–C_7H_7]^+$, 91 (100) $[C_7H_7]^+$; HRMS (EI, 70 eV): calcd for $C_{12}H_{13}{}^{32}S_2$ $[M–C_7H_7]^+$: 221.0459, found 221.0460; IR (ATR, ṽ) = 3027, 2986, 2961, 2907, 2871, 2844, 1493, 1452, 1435, 1239, 1201, 1126, 1068, 1027, 1000, 938, 914, 806, 776, 707, 694, 620, 572, 543, 499, 473, 415 cm^{-1}.

The conditions for this reaction were deposited at Chemotion-repository:
https://dx.doi.org/10.14272/reaction/SA-FUHFF-UHFFFADPSC-GXKYJSRHQM-UHFFFADPSC-NUHFF-NUHFF-NUHFF-ZZZ

Original Data for the target compound can be accessed on the repository Chemotion:

**Type**	**DOI**	**Owner**[1]
**13**	GXKYJSRHQMPAPZ-UHFFFAOYSA-N.1	
1H NMR	https://dx.doi.org/10.14272/GXKYJSRHQMPAPZ-UHFFFAOYSA-N/1HNMR	A
13C NMR	https://dx.doi.org/10.14272/GXKYJSRHQMPAPZ-UHFFFAOYSA-N/13CNMR	A
Mass	https://dx.doi.org/10.14272/GXKYJSRHQMPAPZ-UHFFFAOYSA-N/Mass	A
HRMS	https://dx.doi.org/10.14272/GXKYJSRHQMPAPZ-UHFFFAOYSA-	A

	N/Mass.1	
IR	https://dx.doi.org/10.14272/GXKYJSRHQMPAPZ-UHFFFAOYSA-N/IR	A
**14**	IPSCQEYBZPKANH-UHFFFAOYSA-N	
X-Ray	https://dx.doi.org/10.14272/IPSCQEYBZPKANH-UHFFFAOYSA-N/Crystal-Structure	A

[1] A = data of the authors; B = data provided by others

5.16 1,3-Bis(phenylthio)bicyclo[1.1.1]pentane (**6a**), 1-(Phenylthio)-3-(*p*-phenylthio)bicyclo[1.1.1]pentane (**15**), 1,3-Bis(*p*-tolylthio)bicyclo[1.1.1]pentane (**6d**)

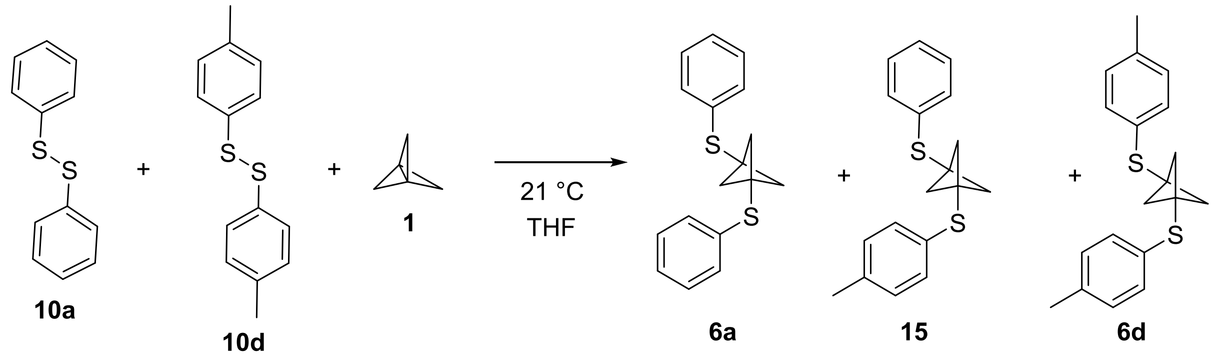

Name {P1|**6a**}: 1,3-Bis(phenylthio)bicyclo[1.1.1]pentane; Formula: $C_{17}H_{16}S_2$; Molecular Mass: 284.4389; Exact Mass: 284.0693;
Smiles: c1ccc(cc1)SC12CC(C1)(C2)Sc1ccccc1;
InChIKey: GHXRODNNSAZUSG-UHFFFAOYSA-N

Name {P2|**15**}: 1-(Phenylthio)-3-(*p*-phenylthio)bicyclo[1.1.1]pentane; Formula: $C_{18}H_{18}S_2$; Molecular Mass: 298.4655; Exact Mass: 298.0850;
Smiles: Cc1ccc(cc1)SC12CC(C1)(C2)Sc1ccccc1;
InChIKey: DWANMFCYEOMCOA-UHFFFAOYSA-N

Name {P3|**6d**}: 1,3-Bis(*p*-tolylthio)bicyclo[1.1.1]pentane; Formula: $C_{19}H_{20}S_2$; Molecular Mass: 312.4921; Exact Mass: 312.1006;
Smiles: Cc1ccc(cc1)SC12CC(C1)(C2)Sc1ccc(cc1)C;
InChIKey: NZNGNSDNZKZZOJ-UHFFFAOYSA-N

According to General Procedure a: {A|**10a**} (Phenyldisulfanyl)benzene (0.137 g, 0.630 mmol, 1.50 equiv); {B|**10d**} 1-Methyl-4-[(4-methylphenyl)disulfanyl]benzene (0.155 g, 0.630 mmol, 1.50 equiv); {C|**1**} [1.1.1]Propellane (0.0277 g, 0.420 mmol, 1.00 equiv); {S1} Tetrahydrofuran (1.0 mL); Yield {P1|**6a**} = 18% (0.0210 g, 0.0738 mmol); {P2|**15**} = 45% (0.0560 g, 0.188 mmol); {P3|**6d**} = 24% (0.0310 g, 0.0992 mmol).
The obtained crude product was purified via flash-chromatography on silica gel using pentane. One fraction with a mixture of the three products was obtained. The yield was determined by NMR spectroscopy. Analytically pure samples were obtained by purification via HPLC (C18, H_2O/ACN 20:80 to 0:100). Product {P1|**6a**}: white solid,

m.p. 54 °C. Product {P2|**15**}: white solid, m.p. 48 °C; R_f {P2|**15**} = 0.11 (pentane). Product {P3|**6d**}: white solid, m.p. 50 °C.

Analysis of {P2|**15**}:

^{1}H NMR (400 MHz, $CDCl_3$, ppm) δ = 7.44–7.40 (m, 2H, Ar-H), 7.33–7.28 (m, 5H, Ar-H), 7.11 (d, *J* = 7.7 Hz, 2H, Ar-H), 2.34 (s, 3H, CH_3), 2.01 (s, 6H, 3xCH_2); ^{13}C NMR (100 MHz, $CDCl_3$, ppm) δ = 138.2 (C_{quat}, $C_{Ar}CH_3$), 134.2 (+, 2xCH_{Ar}), 133.9 (+, 2xCH_{Ar}), 133.4 (C_{quat}, $C_{Ar}S$), 129.8 (+, 2xCH_{Ar}), 129.6 (C_{quat}, $C_{Ar}S$), 123.0 (+, 2xCH_{Ar}), 128.0 (+, CH_{Ar}), 57.4 (–, 3xCH_2), 42.8 (C_{quat}, CCH_2), 42.6 (C_{quat}, CCH_2), 21.3 (+, CH_3); EI (***m/z***, 70 eV, 50 °C): 298 (10) $[M]^+$, 189 (100) $[M–C_6H_5S]^+$, 175 (97) $[M–C_7H_7S]^+$, 91 (27) $[C_7H_7]^+$; HRMS (EI, 70 eV): calcd for $C_{18}H_{18}{}^{32}S_2$ [M]+: 298.0850, found 298.0851; IR (ATR, ṽ) = 2989, 2962, 2908, 2867, 1489, 1473, 1436, 1198, 1176, 1126, 1101, 1092, 1062, 1026, 1016, 926, 809, 779, 744, 703, 690, 554, 511, 480, 421 cm^{-1};

The conditions for this reaction were deposited at Chemotion-repository: https://dx.doi.org/10.14272/reaction/SA-FUHFF-UHFFFADPSC-USTASXYEIS-UHFFFADPSC-NUHFF-NUHFF-NUHFF-ZZZ

Original Data for the target compound can be accessed on the repository Chemotion:

**Type**	**DOI**	**Owner**[1]
**6a**	GHXRODNNSAZUSG-UHFFFAOYSA-N.5	
1H NMR	https://dx.doi.org/10.14272/GHXRODNNSAZUSG-UHFFFAOYSA-N/1HNMR.3	A
**6d**	NZNGNSDNZKZZOJ-UHFFFAOYSA-N.3	
1H NMR	https://dx.doi.org/10.14272/NZNGNSDNZKZZOJ-UHFFFAOYSA-N/1HNMR.2	A
**15**	DWANMFCYEOMCOA-UHFFFAOYSA-N.1	
1H NMR	https://dx.doi.org/10.14272/DWANMFCYEOMCOA-UHFFFAOYSA-N/1HNMR	A
13C NMR	https://dx.doi.org/10.14272/DWANMFCYEOMCOA-UHFFFAOYSA-N/13CNMR	A
Mass	https://dx.doi.org/10.14272/DWANMFCYEOMCOA-UHFFFAOYSA-N/Mass	A
HRMS	https://dx.doi.org/10.14272/DWANMFCYEOMCOA-UHFFFAOYSA-N/Mass.1	A
IR	https://dx.doi.org/10.14272/DWANMFCYEOMCOA-UHFFFAOYSA-N/IR	A

[1] A = data of the authors; B = data provided by others

5.17 1,3-Bis(phenylthio)bicyclo[1.1.1]pentane (**6a**), 1-(Phenylthio)-3-(4-methoxyphenylthio)bicyclo[1.1.1]pentane (**16**), 1,3-Bis((4-methoxyphenyl)thio)bicyclo[1.1.1]pentane (**6e**)

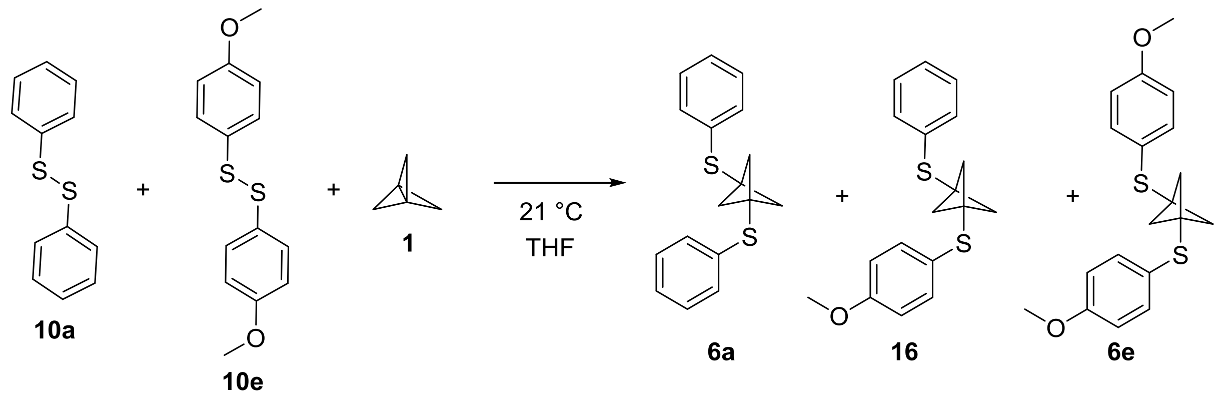

Name {P1|**6a**}: 1,3-Bis(phenylthio)bicyclo[1.1.1]pentane; Formula: $C_{17}H_{16}S_2$; Molecular Mass: 284.4389; Exact Mass: 284.0693;
Smiles: c1ccc(cc1)SC12CC(C1)(C2)Sc1ccccc1;
InChIKey: GHXRODNNSAZUSG-UHFFFAOYSA-N

Name {P2|**16**}: 1-(Phenylthio)-3-((4-methoxyphenyl)thio)bicyclo[1.1.1]pentane; Formula: $C_{18}H_{18}OS_2$; Molecular Mass: 314.4649; Exact Mass: 314.0799;
Smiles: COc1ccc(cc1)SC12CC(C1)(C2)Sc1ccccc1;
InChIKey: MMODRWTWUUODQN-UHFFFAOYSA-N

Name {P3|**6e**}: 1,3-Bis((4-methoxyphenyl)thio)bicyclo[1.1.1]pentane; Formula: $C_{19}H_{20}O_2S_2$; Molecular Mass: 344.4909; Exact Mass: 344.0905;
Smiles: COc1ccc(cc1)SC12CC(C1)(C2)Sc1ccc(cc1)OC;
InChIKey: AWYXHOMVLAPYSJ-UHFFFAOYSA-N

According to General Procedure a: {A|**10a**} (Phenyldisulfanyl)benzene (0.143 g, 0.655 mmol, 1.50 equiv); {B|**10e**} Bis(4-methoxyphenyl) disulfide (0.182 g, 0.655 mmol, 1.50 equiv); {C|**1**} [1.1.1]Propellane (0.0289 g, 0.437 mmol, 1.00 equiv); {S1} Tetrahydrofuran (1.0 mL); Yield {P1|**6a**} = 18% (22.0 mg, 0.0773 mmol); {P2|**16**} = 49% (0.0670 g, 0.213 mmol); {P3|**6e**} = 31% (0.0460 g, 0.134 mmol).
The obtained crude product was purified *via* flash-chromatography on silica gel using pentane to pentane/diethyl ether 50:1. Mixed fractions were collected and purified by preparative TLC using pentane/diethyl ether 50:1. Product {P1|**6a**}: white solid, m.p. 54 °C. Product {P2|**16**}: white solid, m.p. 71 °C; R_f {P2|**16**} = 0.33 (pentane/diethyl ether 100:1). Product {P3|**6e**}: white solid, m.p. 66 °C.
Analysis of {P2|**16**}:
^{1}H NMR (500 MHz, $CDCl_3$, ppm) δ = 7.41–7.37 (m, 2H, Ar-H), 7.36–7.32 (m, 2H, Ar-H), 7.31–7.27 (m, 3H, Ar-H), 6.85–6.81 (m, 2H, Ar-H), 3.80 (s, 3H, CH_3), 1.96 (s, 6H, 3xCH_2); ^{13}C NMR (125 MHz, $CDCl_3$, ppm) δ = 159.8 (C_{quat}, C_{Ar}O), 136.2 (+, 2xCH_{Ar}), 133.8 (+, 2xCH_{Ar}), 133.3 (C_{quat}, C_{Ar}S), 128.9 (+, 2xCH_{Ar}), 127.9 (+, CH_{Ar}), 123.5 (C_{quat}, C_{Ar}S), 114.5 (+, 2xCH_{Ar}), 57.1 (–, 3xCH_2), 55.3 (+, CH_3), 43.0 (C_{quat}, CCH_2), 42.4 (C_{quat}, CCH_2); EI (***m/z***, 70 eV, 80 °C): 314 (20) $[M]^+$, 205 (100) $[M–C_6H_5S]^+$, 175 (89) $[M–C_7H_7OS]^+$, 139 (76) $[C_7H_7OS]^+$; HRMS (EI, 70 eV): calcd for $C_{18}H_{18}O^{32}S_2$ [M]+: 314.0799, found 314.0798; IR (ATR, ṽ) = 2989, 2956, 2907, 2867, 2834, 1588, 1570, 1489, 1458, 1438, 1404, 1282, 1237, 1197, 1170, 1126, 1095, 1064, 1027, 1006, 928, 833, 816, 798, 779, 747, 688, 640, 554, 527, 499, 486, 428, 388 cm^{-1}.

The conditions for this reaction were deposited at Chemotion-repository:

https://dx.doi.org/10.14272/reaction/SA-FUHFF-UHFFFADPSC-MRDPMFJFXN-UHFFFADPSC-NUHFF-NUHFF-NUHFF-ZZZ

Original Data for the target compound can be accessed on the repository Chemotion:

Type	DOI	Owner[1]
**6a**	GHXRODNNSAZUSG-UHFFFAOYSA-N	
1H NMR	https://dx.doi.org/10.14272/GHXRODNNSAZUSG-UHFFFAOYSA-N/1HNMR.2	A
**6e**	AWYXHOMVLAPYSJ-UHFFFAOYSA-N	
1H NMR	https://dx.doi.org/10.14272/AWYXHOMVLAPYSJ-UHFFFAOYSA-N/1HNMR.1	A
**16**	MMODRWTWUUODQN-UHFFFAOYSA-N	
1H NMR	https://dx.doi.org/10.14272/MMODRWTWUUODQN-UHFFFAOYSA-N/1HNMR	A
13C NMR	https://dx.doi.org/10.14272/MMODRWTWUUODQN-UHFFFAOYSA-N/13CNMR	A
Mass	https://dx.doi.org/10.14272/MMODRWTWUUODQN-UHFFFAOYSA-N/Mass	A

HRMS	https://dx.doi.org/10.14272/MMODRWTWUUODQN-UHFFFAOYSA-N/Mass.1	A
IR	https://dx.doi.org/10.14272/MMODRWTWUUODQN-UHFFFAOYSA-N/IR	A
X-Ray	https://dx.doi.org/10.14272/MMODRWTWUUODQN-UHFFFAOYSA-N/Crystal-Structure	A

[1] A = data of the authors; B = data provided by others

6 Crystallographic information

Crystal Structure Determination of 6a

The single-crystal X-ray diffraction study was carried out on a Bruker D8 Venture diffractometer with PhotonII CPAD detector at 123(2) K using Cu-Ka radiation (*l* = 1.54178 Å). Dual space methods (SHELXT) [G. M. Sheldrick, *Acta Crystallogr.* 2015, **A71**, 3-8] were used for structure solution and refinement was carried out using SHELXL-2014 (full-matrix least-squares on F^2) [G. M. Sheldrick, *Acta Crystallogr.* 2015, **C71**, 3-8]. Hydrogen atoms were localized by difference electron density determination and refined using a riding model. A semi-empirical absorption corrections was applied. The absolute structure was determined by refinement of Parsons' x-parameter [S. Parson, H. D. Flack, T. Wagner, *Acta Crystallogr.* 2013, **B69**, 249-259]

6a: colourless crystals, $C_{17}H_{16}S_2$, M_r = 284.42, crystal size 0.36 × 0.16 × 0.06 mm, monoclinic, space group $P2_1$ (no. 4), a = 9.7073(3) Å, b = 5.6219(2) Å, c = 14.3212(5) Å, β = 109.485(1)°, V = 736.80(4) Å^3, Z = 2, ρ = 1.282 Mg/m^{-3}, μ(Cu-K$_\alpha$) = 3.12 mm^{-1}, F(000) = 300, $2\theta_{max}$ = 144.2°, 14308 reflections, of which 2772 were independent (R_{int} = 0.030), 172 parameters, 1 restraint, R_1 = 0.026 (for 2763 I > 2σ(I)), wR_2 = 0.070 (all data), S = 1.04, largest diff. peak / hole = 0.32 / -0.17 e Å^{-3}, x = -0.11(12).

CCDC 1896780 (**6a**) contains the supplementary crystallographic data for this paper. These data can be obtained free of charge from The Cambridge Crystallographic Data Centre via www.ccdc.cam.ac.uk/data_request/cif.

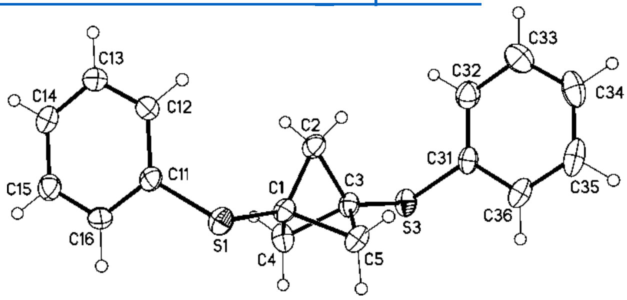

Figure S1: Molecular structure of **6a** (displacement parameters are drawn at 50% probability level).

Crystal structure determination of 14

Single crystal X-ray diffraction data of **14** were collected on a STOE STADI VARI diffractometer with monochromated Ga Kα (1.34143 Å) radiation at 200 K. Using Olex2 [1], the structure was solved with the ShelXS [2] structure solution program using Direct Methods and refined with the ShelXL [3] refinement package using Least Squares minimization. Refinement was performed with anisotropic temperature factors for all non-hydrogen atoms; hydrogen atoms were calculated on idealized positions.

14: $C_{24}H_{26}S_2$ (M =378.57 g/mol): monoclinic, space group $P2_1/n$ (no. 14), a = 6.2148(9) Å, b = 11.2735(10) Å, c = 15.165(2) Å, β = 99.751(12)°, V = 1047.1(2) Å^3, Z = 2, T = 200.15 K, μ(GaKα) = 1.506 mm^{-1}, $Dcalc$ = 1.201 g/cm^3, 7376 reflections measured (19.89° ≤ 2Θ ≤ 115.066°), 2112 unique (R_{int} = 0.0339, R_{sigma} = 0.0365) which were used in all calculations. The final R_1 was 0.0443 (I > 2σ(I)) and wR_2 was 0.1296 (all data).
CCDC 1896794 (**14**) contains the supplementary crystallographic data for this paper. These data can be obtained free of charge from The Cambridge Crystallographic Data Centre via www.ccdc.cam.ac.uk/data_request/cif.

[1] O.V. Dolomanov, L.J. Bourhis, R.J. Gildea, J.A.K. Howard, H. Puschmann, *J. Appl. Cryst.* **2009**, *42*, 339–341.
[2] G.M. Sheldrick, *Acta Cryst. A* **2008**, *A64*, 112–122.
[3] G.M. Sheldrick, *Acta Cryst. C* **2015**, *C71*, 3–8.

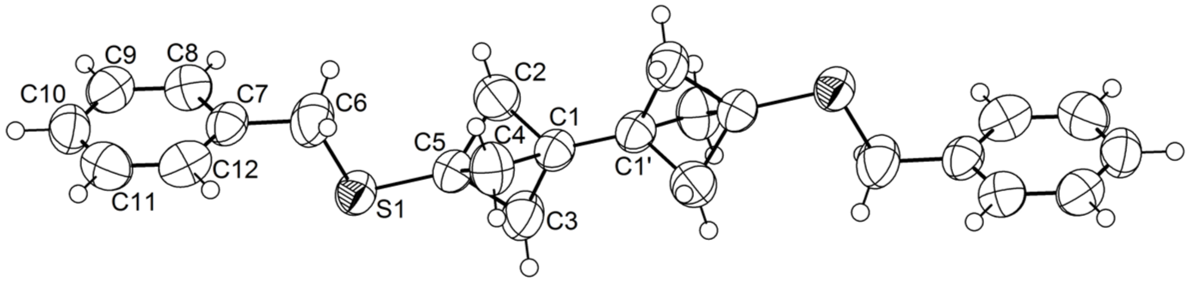

Figure S2: Molecular structure of **14** (displacement parameters are drawn at 50% probability level).

Crystal Structure Determination of 16

The single-crystal X-ray diffraction study was carried out on a Bruker D8 Venture diffractometer with PhotonII CPAD detector at 123(2) K using Cu-Ka radiation (l = 1.54178 Å). Dual space methods (SHELXT) [G. M. Sheldrick, *Acta Crystallogr.* 2015, **A71**, 3-8] were used for structure solution and refinement was carried out using SHELXL-2014 (full-matrix least-squares on F^2) [G. M. Sheldrick, *Acta Crystallogr.* 2015, **C71**, 3-8]. Hydrogen atoms were localized by difference electron density determination and refined using a riding model. A semi-empirical absorption correction was applied. The absolute structure was determined by refinement of Parsons' x-parameter [S. Parson, H. D. Flack, T. Wagner, *Acta Crystallogr.* 2013, **B69**, 249-259]
16: colourless crystals, $C_{18}H_{18}OS_2$, M_r = 314.44, crystal size 0.32 × 0.16 × 0.08 mm, monoclinic, space group $P2_1$ (no. 4), a = 9.6338(5) Å, b = 5.6921(3) Å, c = 14.5108(7) Å, β = 97.628(1)°, V = 788.68(7) Å^3, Z = 2, ρ = 1.324 Mg/m^{-3}, μ(Cu-K_α) = 3.01 mm^{-1}, F(000) = 332, $2\theta_{max}$ = 144.4°, 13068 reflections, of which 3071 were independent (R_{int} = 0.027), 191 parameters, 1 restraint, R_1 = 0.024 (for 3059 I > 2σ(I)), wR_2 = 0.063 (all data), S = 1.04, largest diff. peak / hole = 0.31 / -0.14 e Å^{-3}, x = 0.003(7).

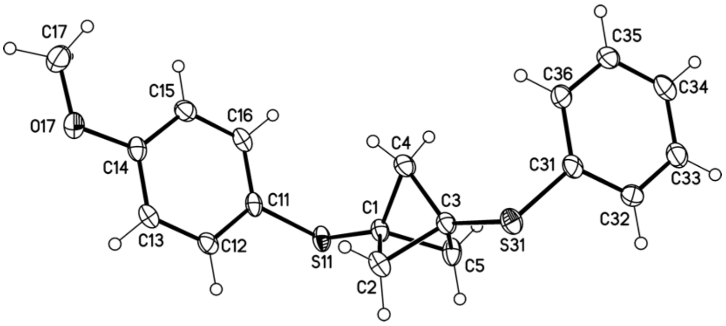

Figure S3: Molecular structure of **16** (displacement parameters are drawn at 50% probability level).
