# Supplementary material for: Insertion of [1.1.1]propellane into aromatic disulfides
Source: Beilstein J Org Chem. 2019 May 28;15:1172–80. doi: 10.3762/bjoc.15.114 (PMC6604700; doi:10.3762/bjoc.15.114)

BEILSTEIN JOURNAL OF ORGANIC CHEMISTRY

Supporting Information

for

Insertion of [1.1.1]propellane into aromatic disulfides

Robin M. Bär, Gregor Heinrich, Martin Nieger, Olaf Fuhr and Stefan Bräse

Beilstein J. Org. Chem. **2019,** *15,* 1172–1180. doi:10.3762/bjoc.15.114

Spectra

License and Terms: This is a supporting information file under the terms of the Creative Commons Attribution License (http://creativecommons.org/licenses/by/4.0). Please note that the reuse, redistribution and reproduction in particular requires that the authors and source are credited.
The license is subject to the *Beilstein Journal of Organic Chemistry* terms and conditions: (https://www.beilstein-journals.org/bjoc)

[**5a**] 3-Phenylsulfanylbicyclo[1.1.1]pentane

^{1}H NMR

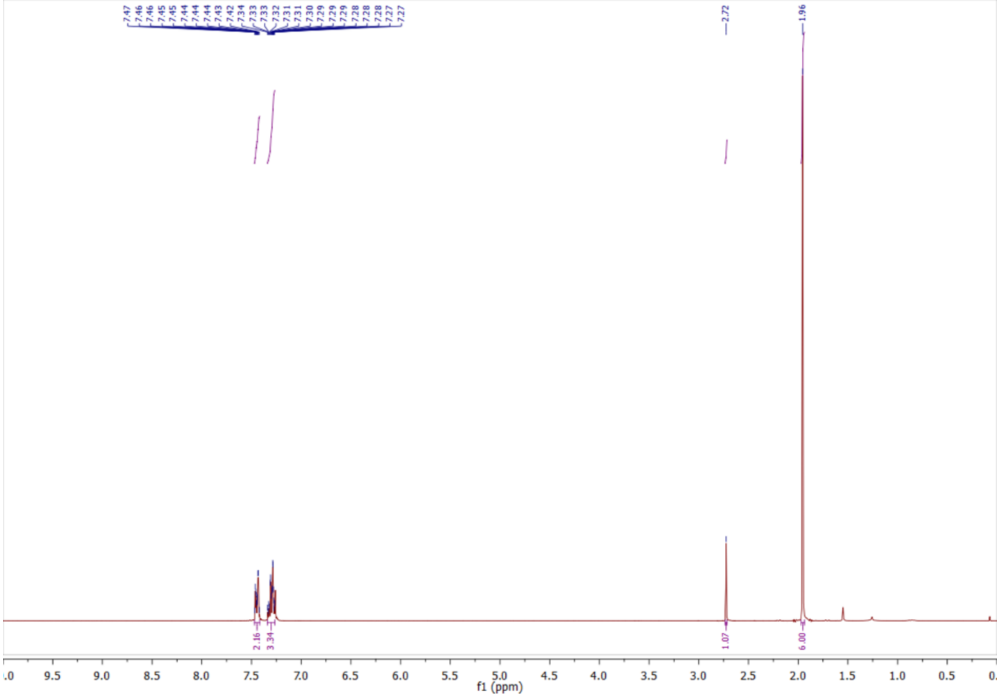

^{13}C NMR

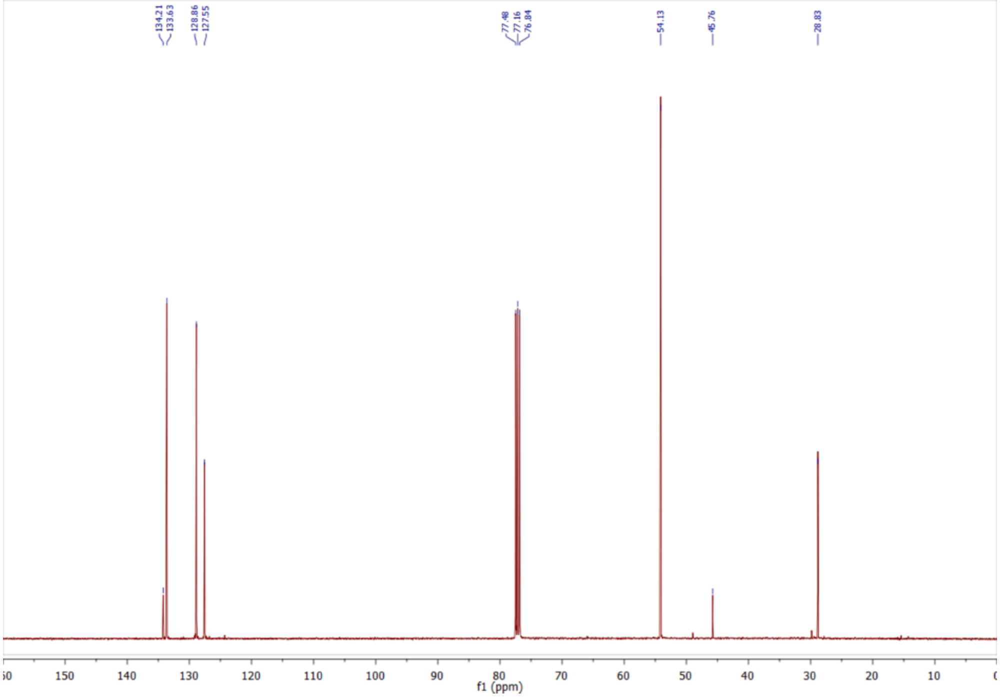

[**6a**] 1,3-Bis(phenylthio)bicyclo[1.1.1]pentane

^{1}H NMR

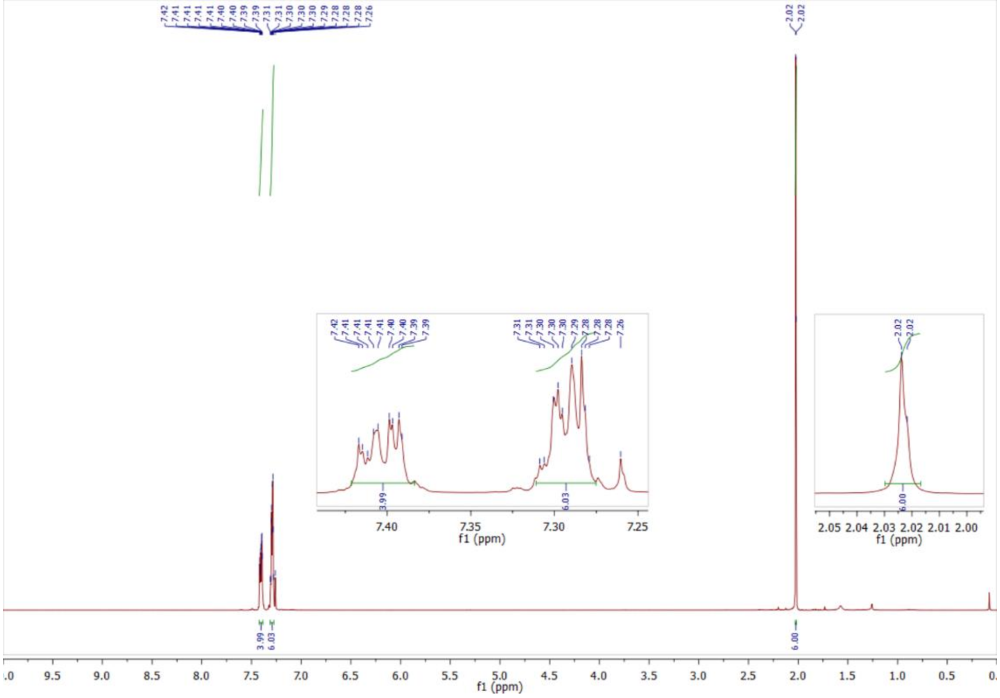

^{13}C NMR

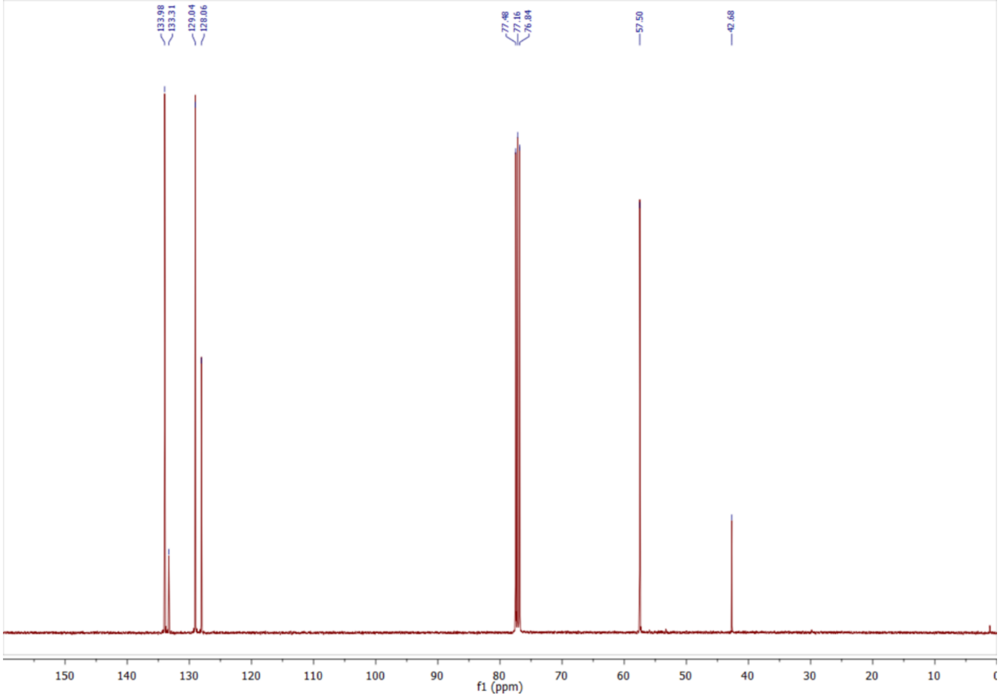

[**6b**] 1,3-Bis((4-chlorophenyl)thio)bicyclo[1.1.1]pentane

^{1}H NMR

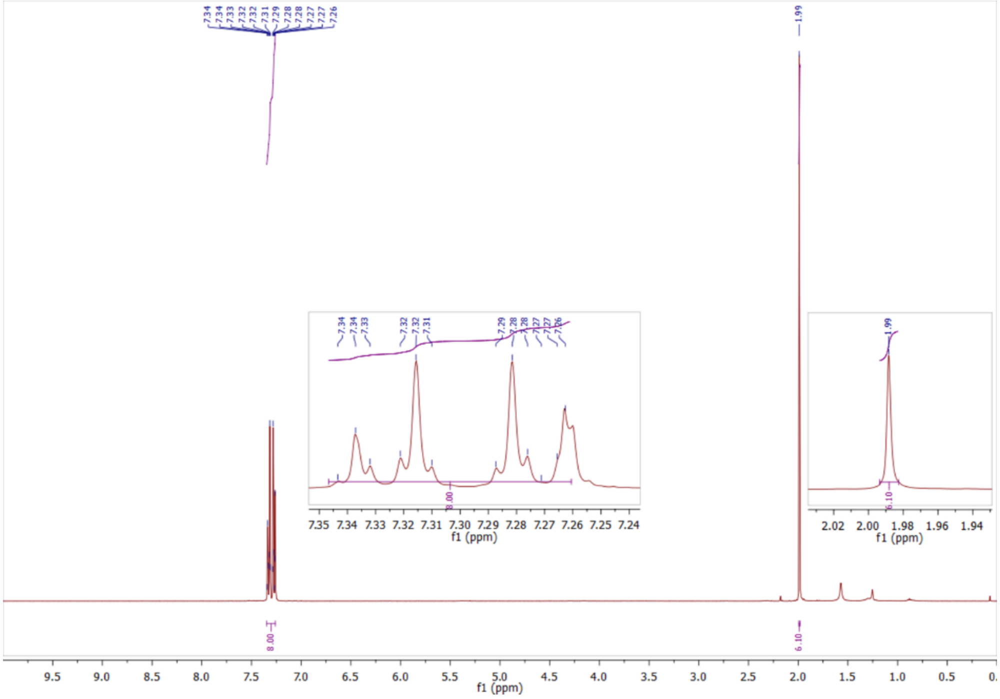

^{13}C NMR

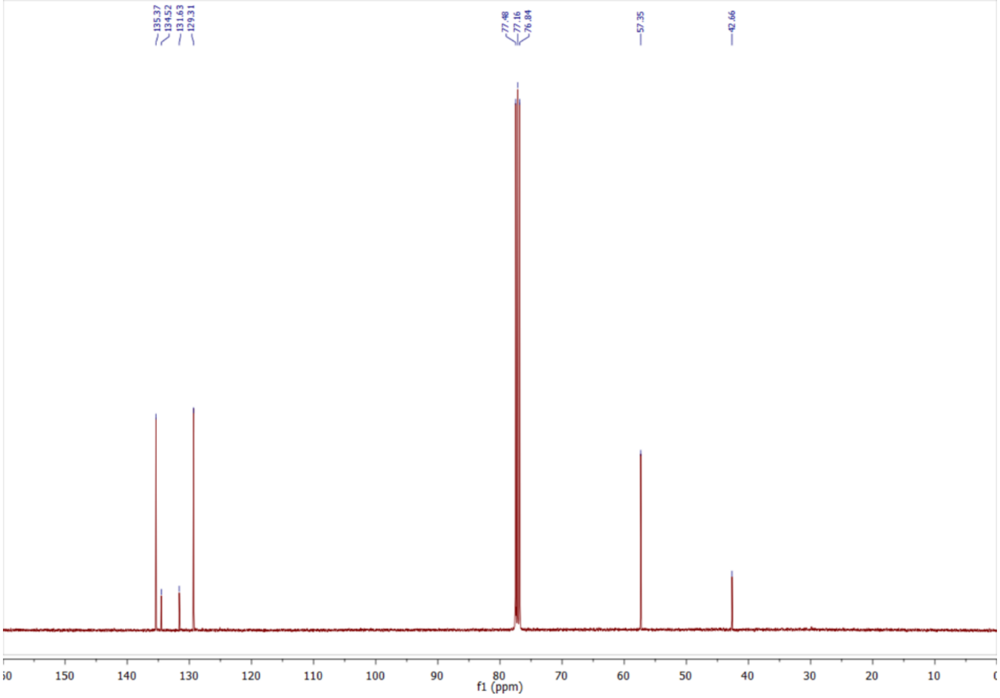

[**6c**] 1,3-Bis((3,5-dichlorophenyl)thio)bicyclo[1.1.1]pentane

^{1}H NMR

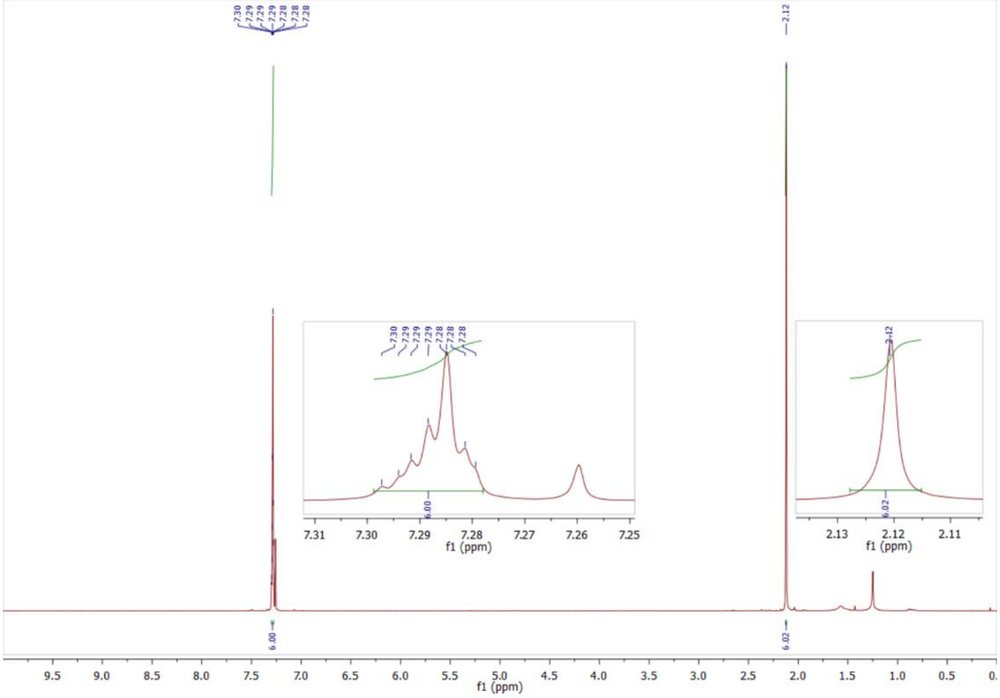

^{13}C NMR

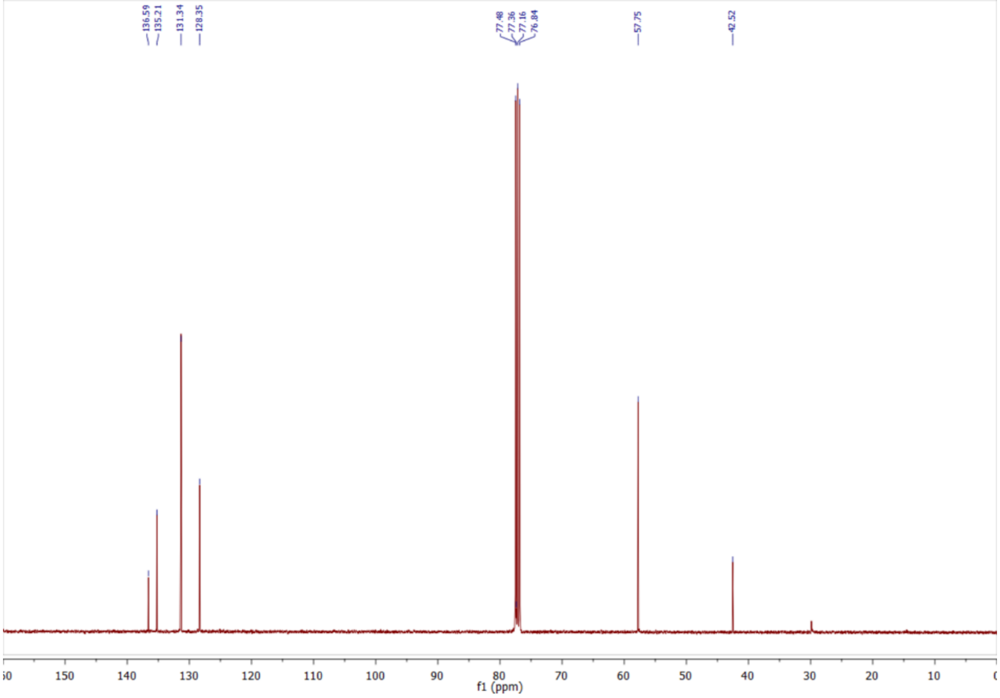

[**6d**] 1,3-Bis(*p*-tolylthio)bicyclo[1.1.1]pentane

^{1}H NMR

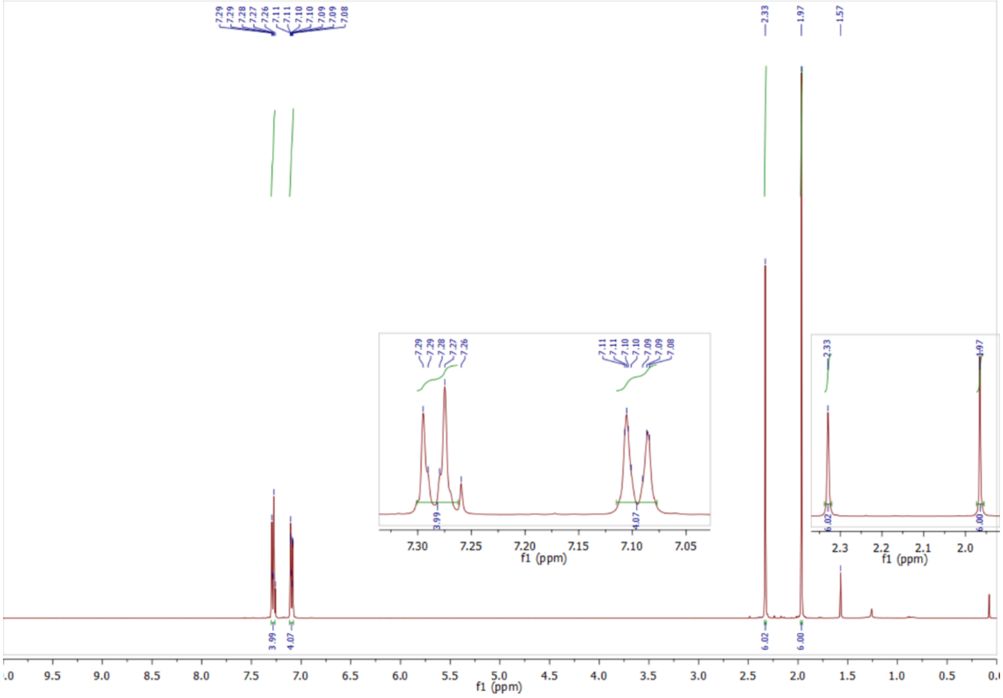

^{13}C NMR

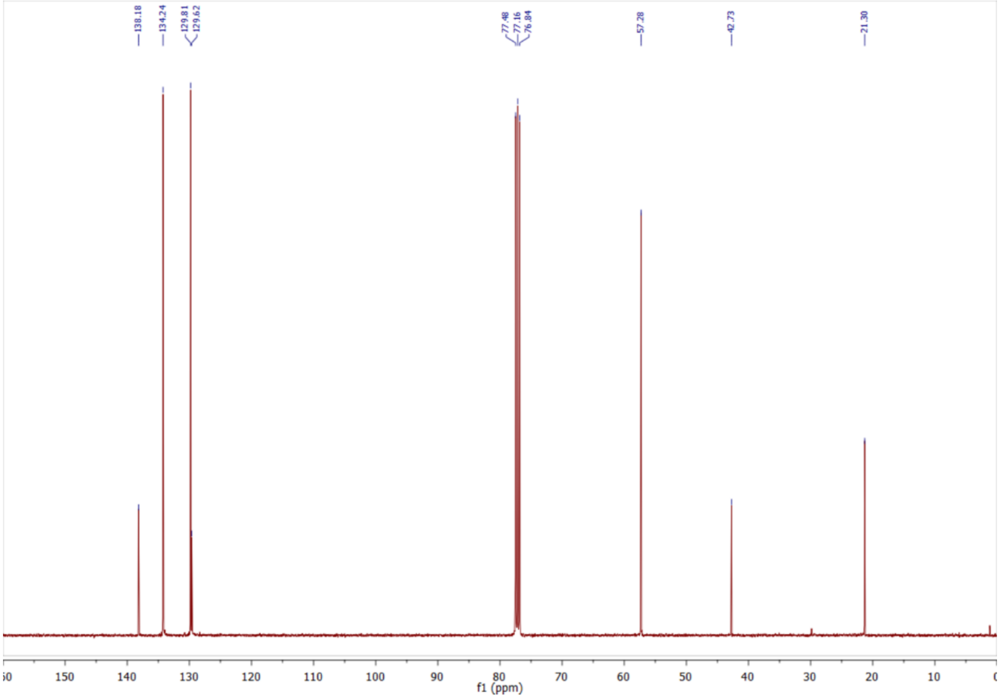

[**6e**] 1,3-Bis((4-methoxyphenyl)thio)bicyclo[1.1.1]pentane

^{1}H NMR

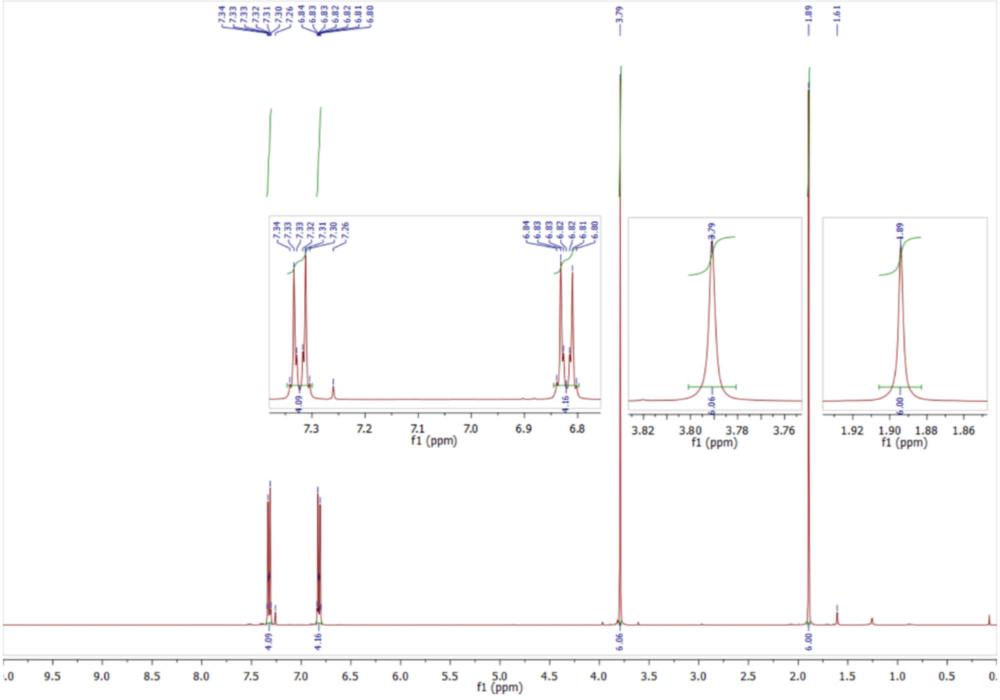

^{13}C NMR

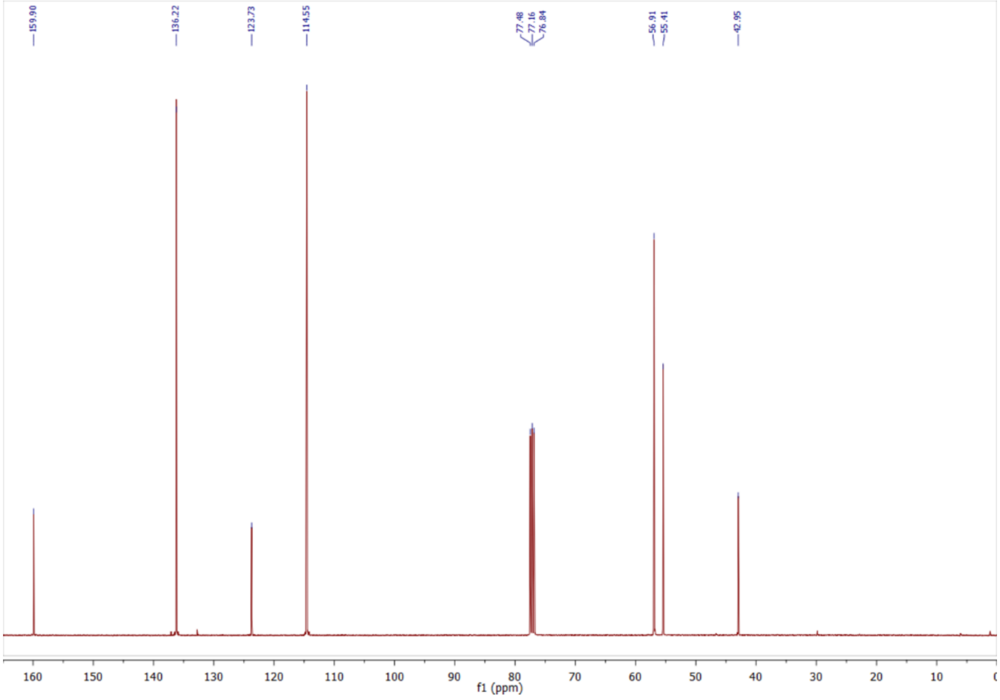

[**6f**] 1,3-Bis([1,1'-biphenyl]-2-ylthio)bicyclo[1.1.1]pentane

^{1}H NMR

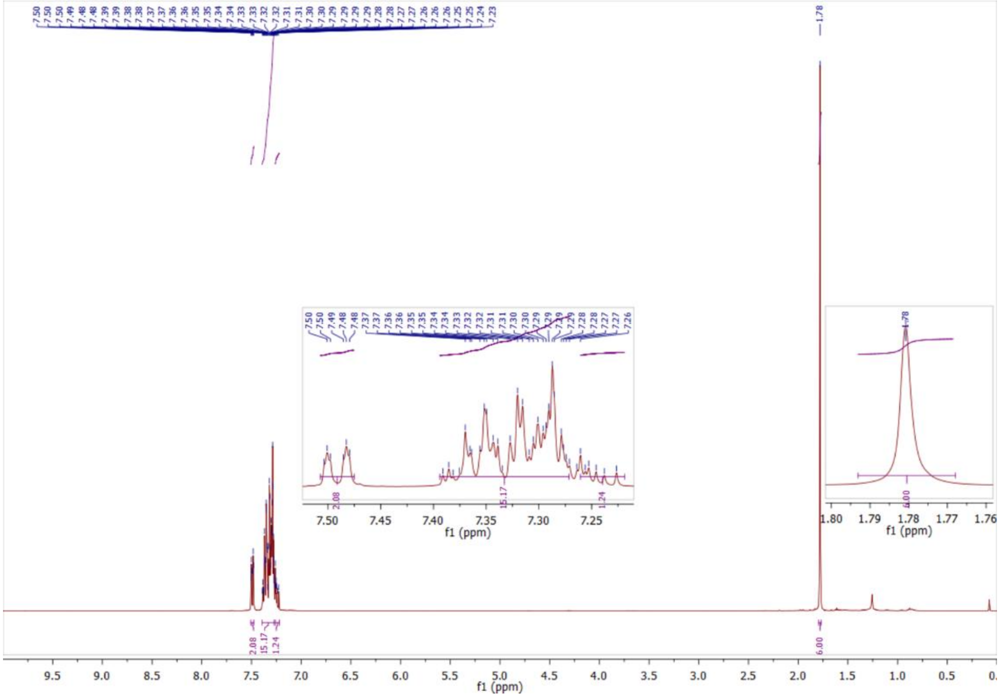

^{13}C NMR

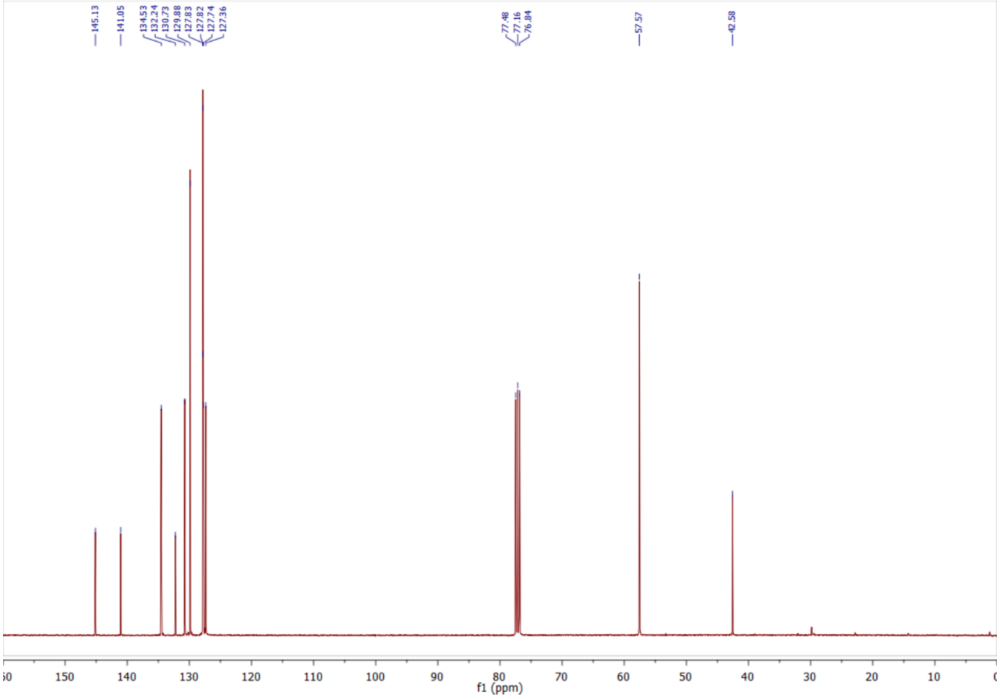

[**10f**] 1-Phenyl-2-[(2-phenylphenyl)disulfanyl]benzene

^{1}H NMR

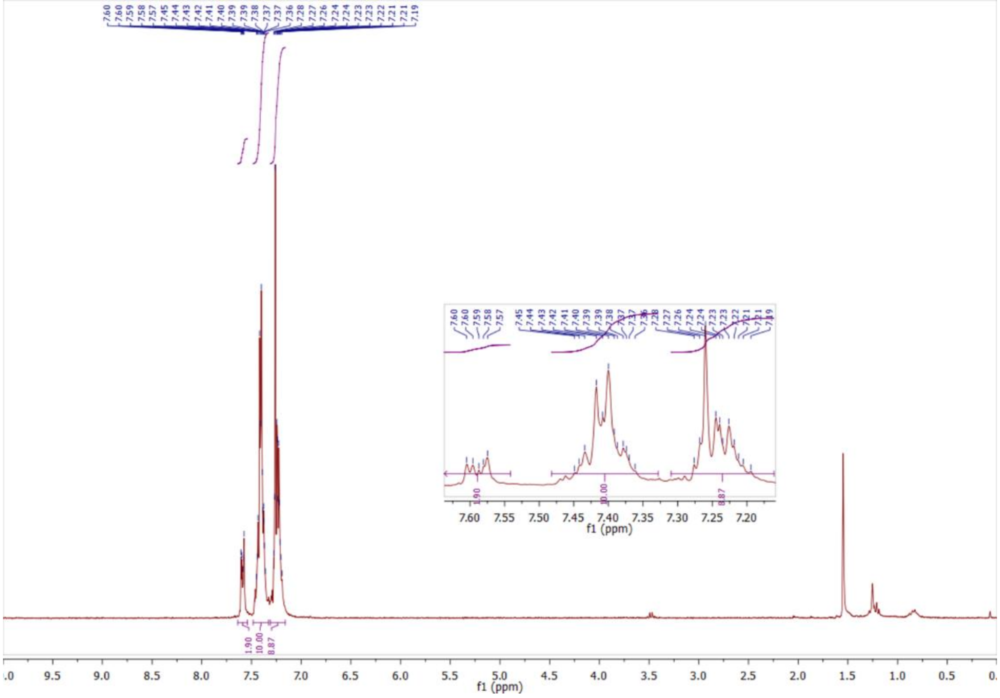

^{13}C NMR

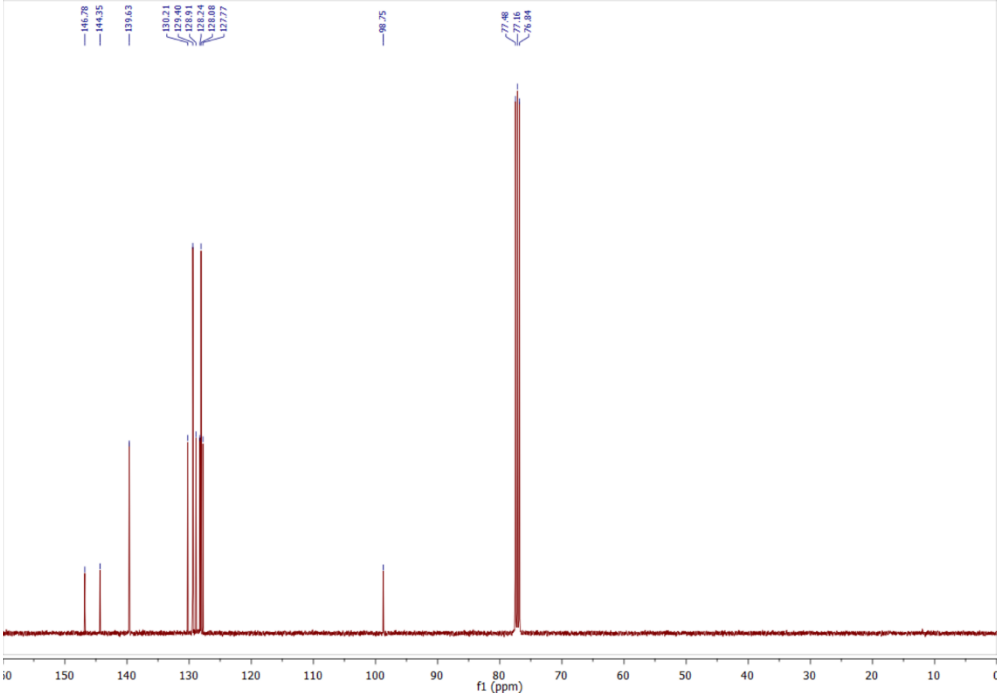

[**11a**] 3,3'-Bis(phenylthio)-1,1'-bi(bicyclo[1.1.1]pentane)

^{1}H NMR

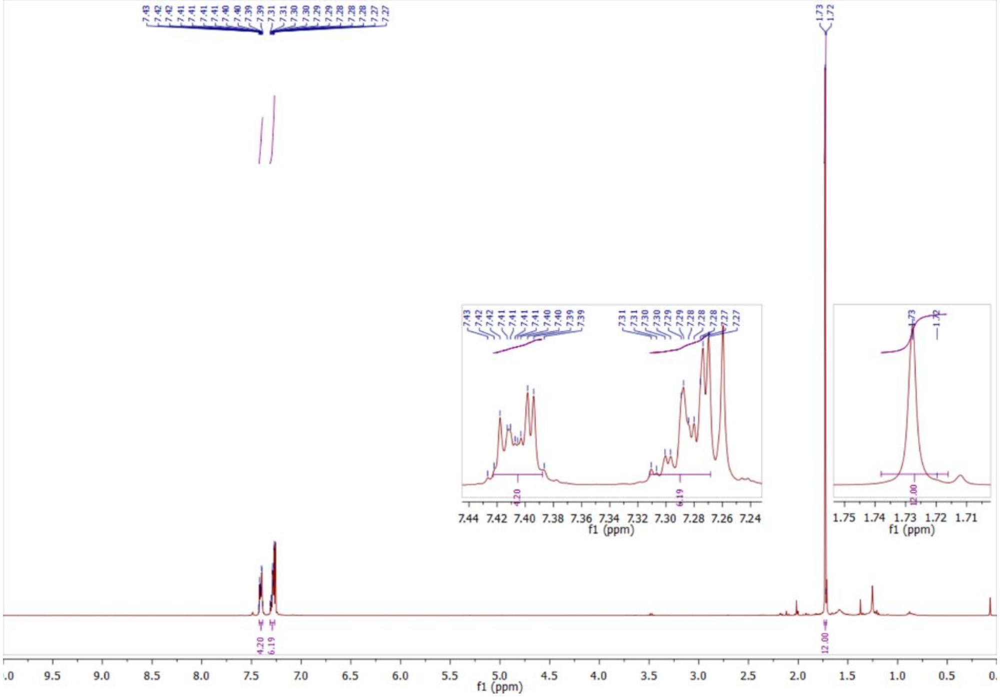

^{13}C NMR

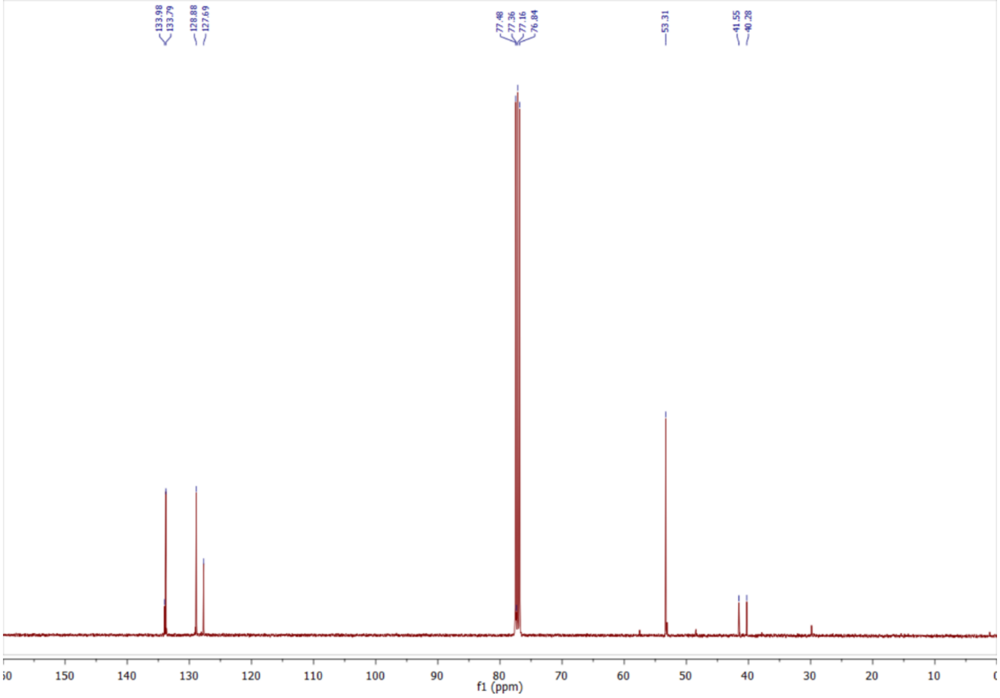

[**11b**] 3,3'-Bis((4-chlorophenyl)thio)-1,1'-bi(bicyclo[1.1.1]pentane)

^{1}H NMR

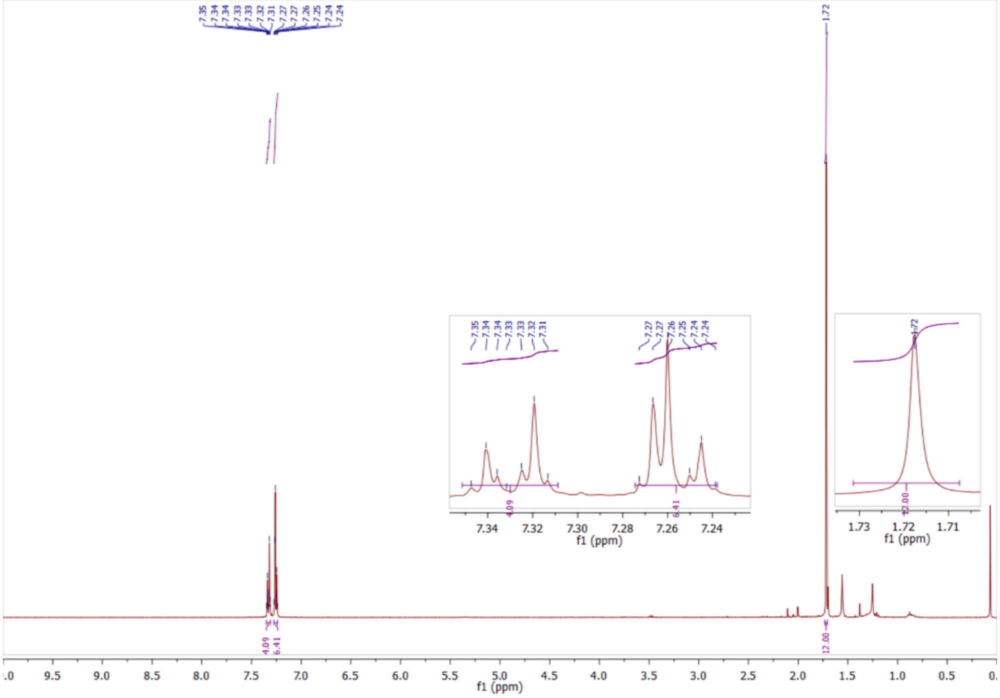

^{13}C NMR

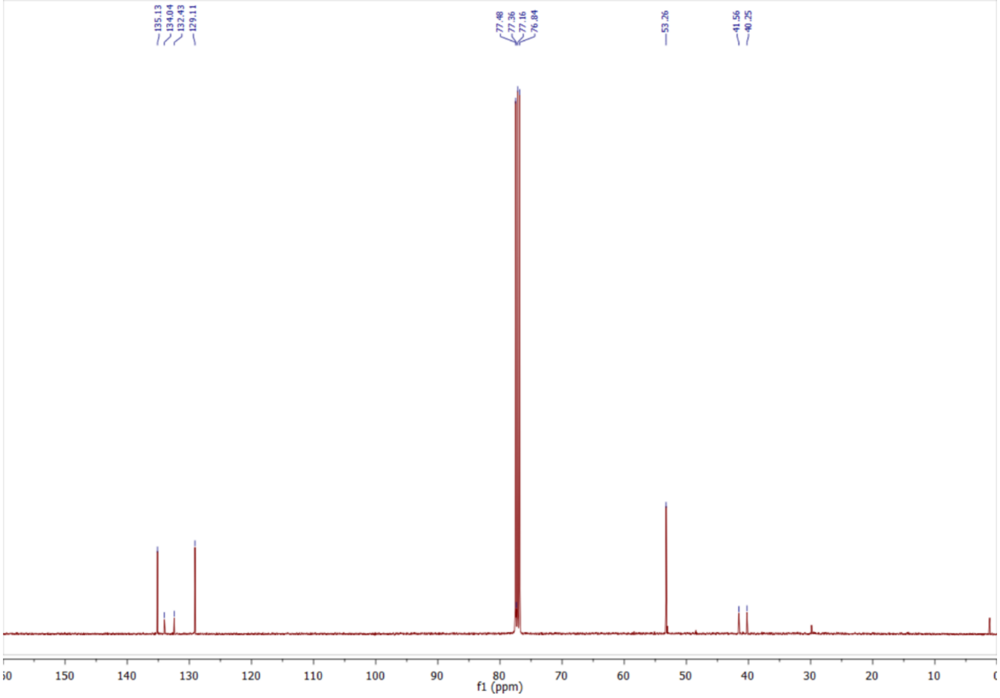

[**11c**] 3,3'-Bis((3,5-dichlorophenyl)thio)-1,1'-bi(bicyclo[1.1.1]pentane)

^{1}H NMR

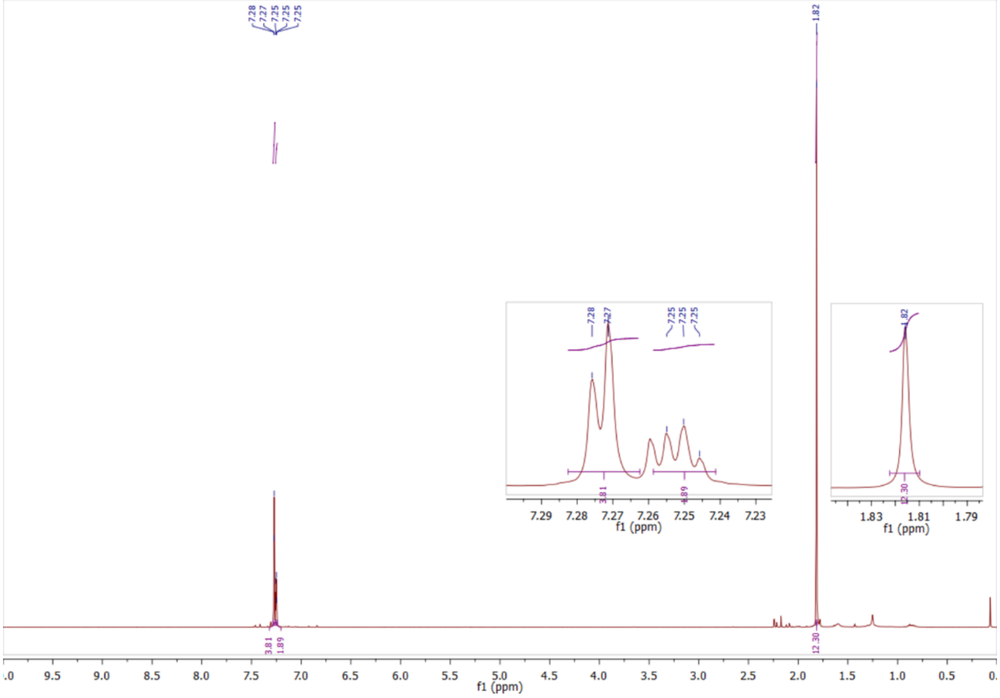

^{13}C NMR

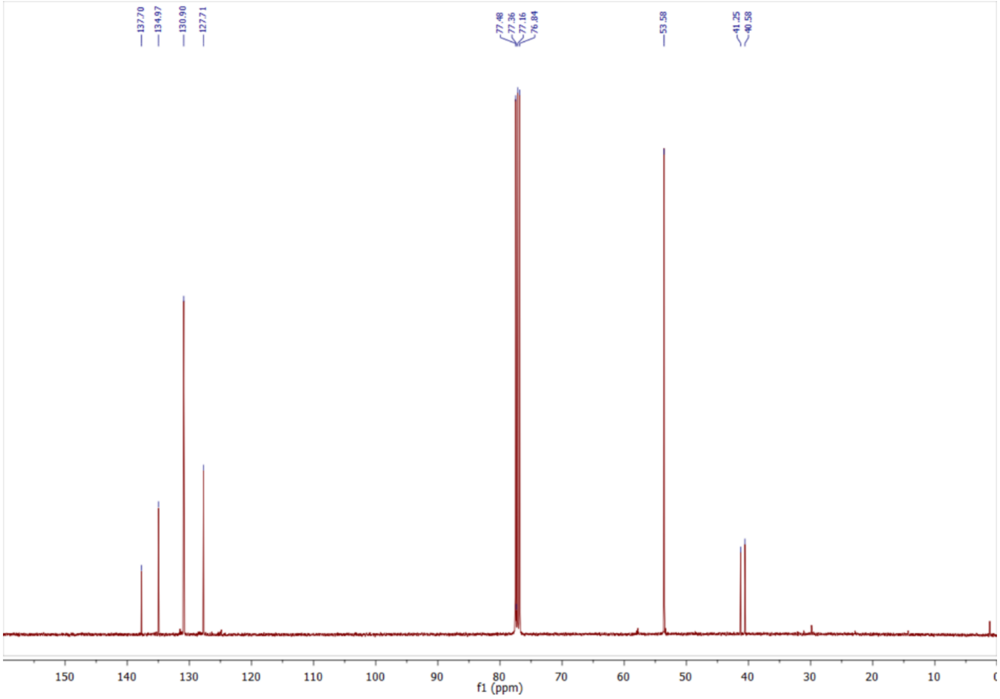

[**11d**] 3,3'-Bis(*p*-tolylthio)-1,1'-bi(bicyclo[1.1.1]pentane)

^{1}H NMR

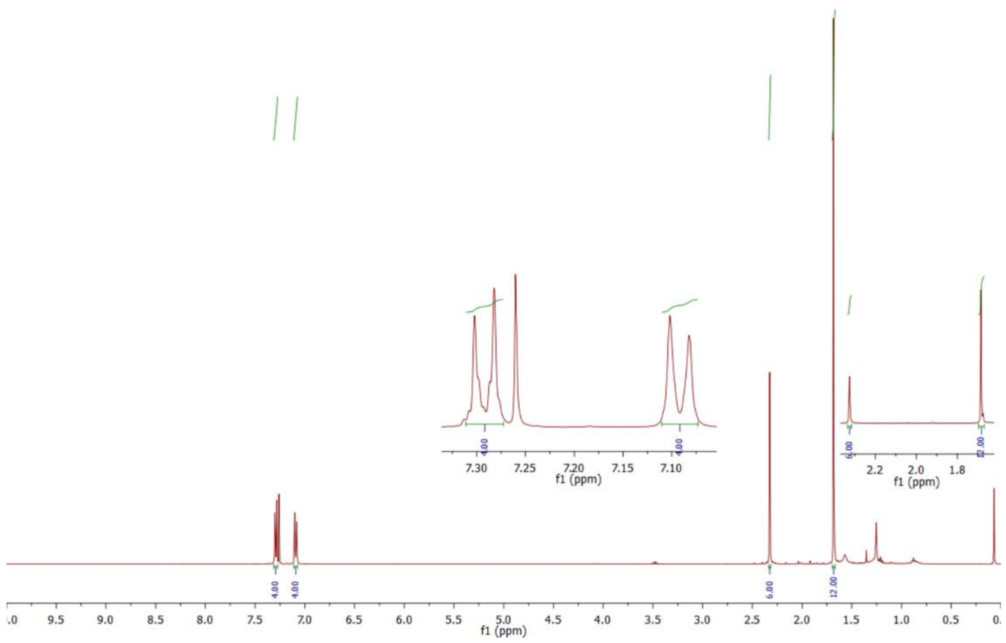

^{13}C NMR

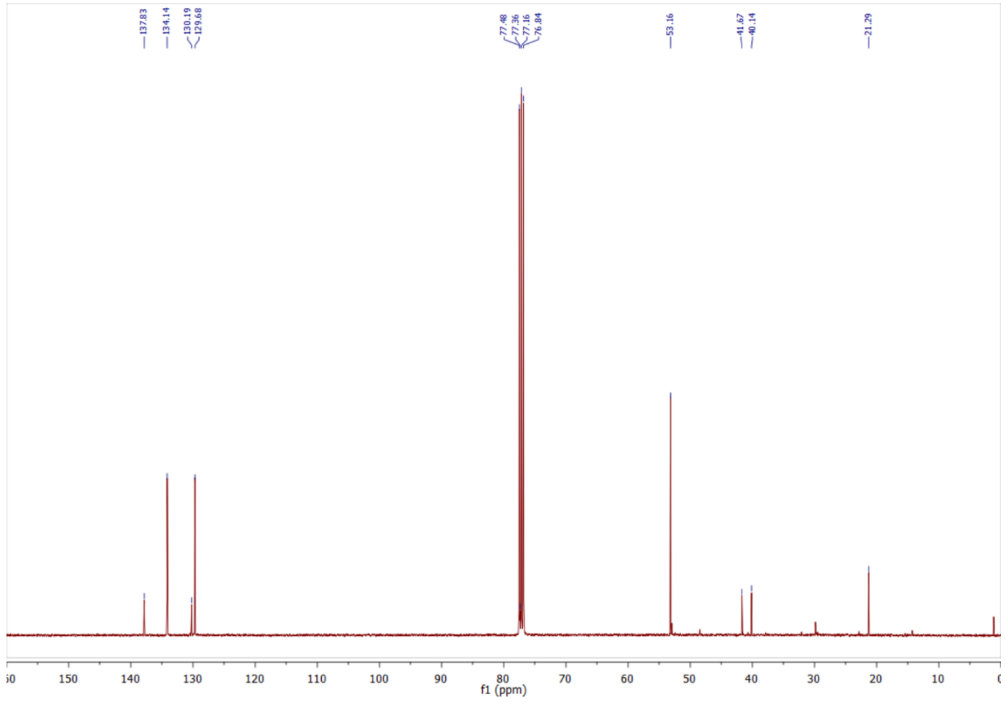

[**13**] 1,3-Bis(benzylthio)bicyclo[1.1.1]pentane

^{1}H NMR

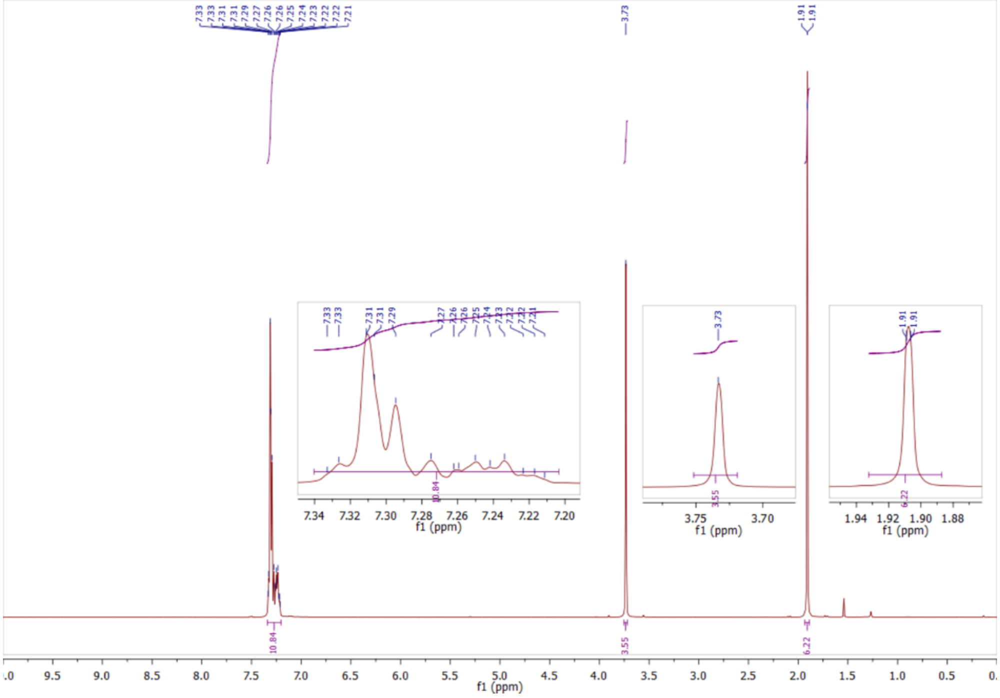

^{13}C NMR

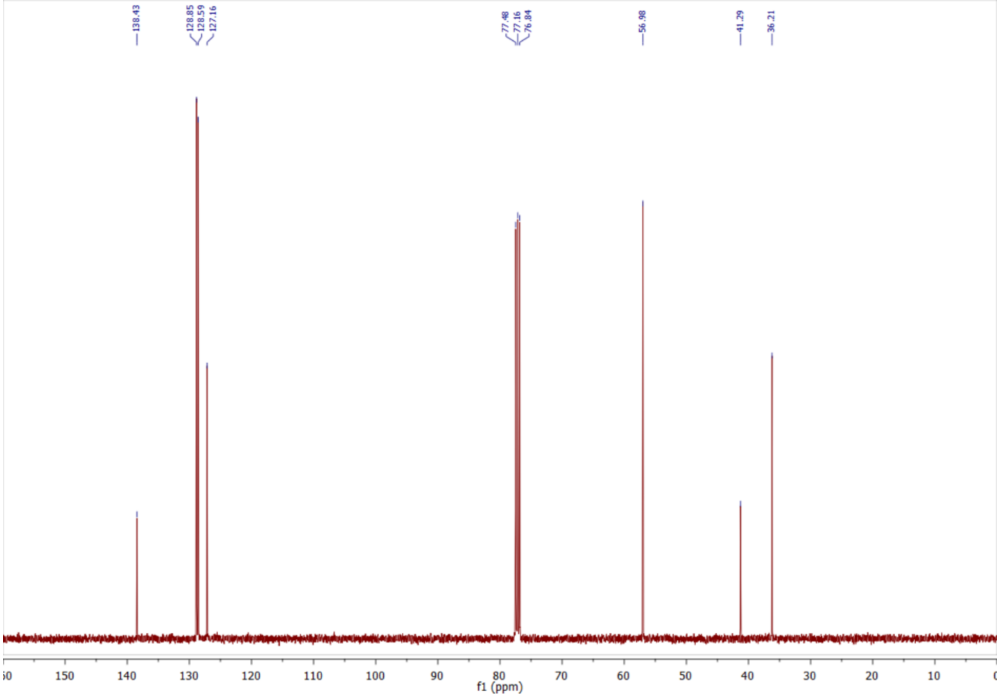

[**15**] 1-(Phenylthio)-3-(*p*-phenylthio)bicyclo[1.1.1]pentane

^{1}H NMR

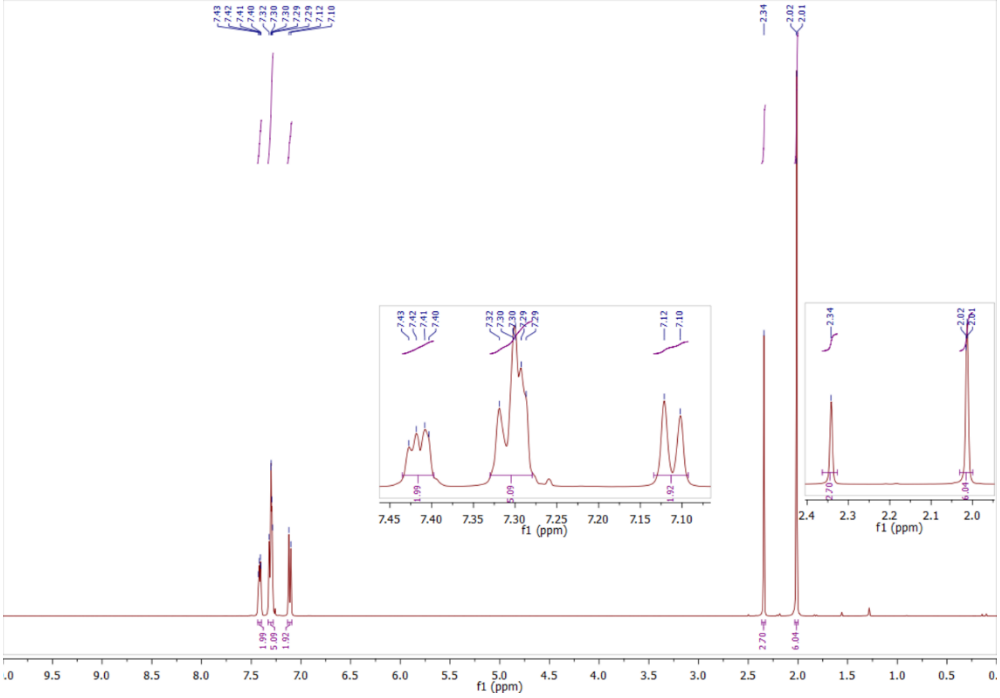

^{13}C NMR

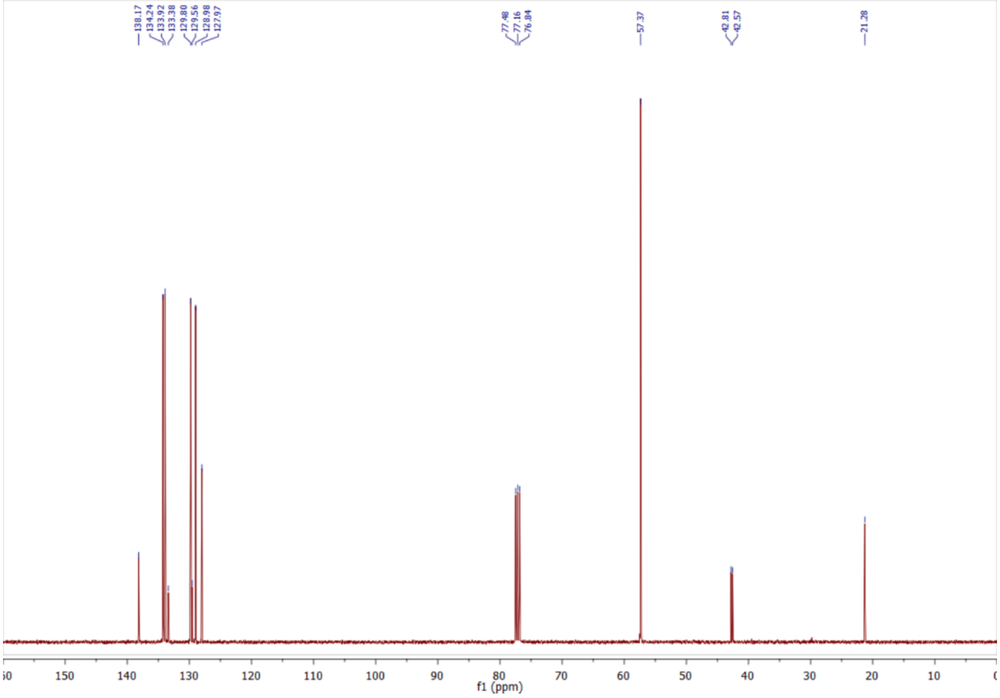

[**16**] 1-(Phenylthio)-3-(4-methoxyphenylthio)bicyclo[1.1.1]pentane

^{1}H NMR

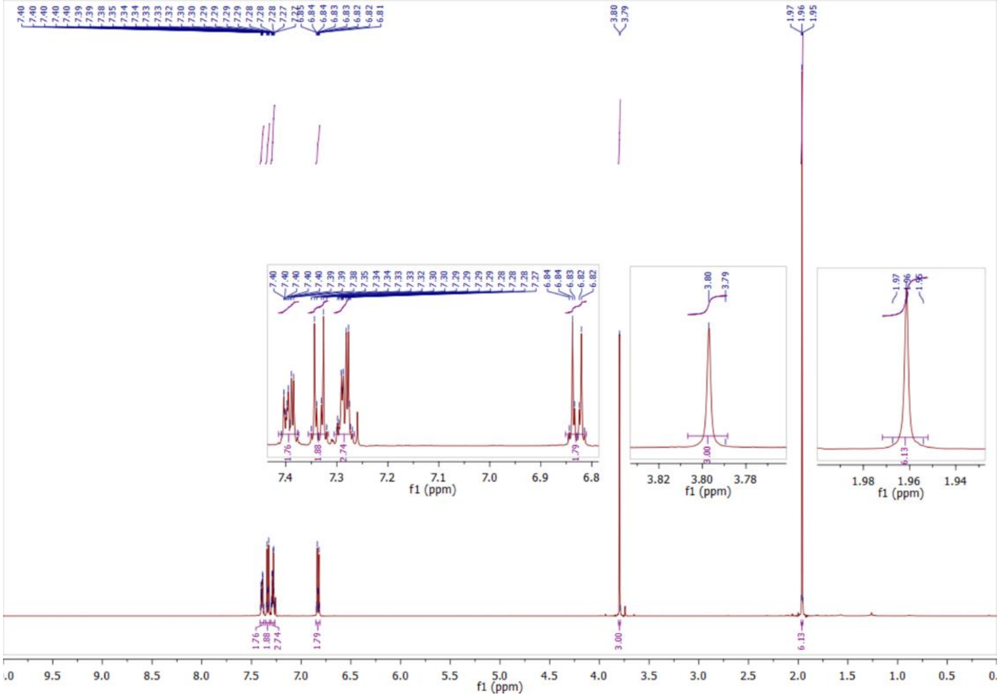

^{13}C NMR

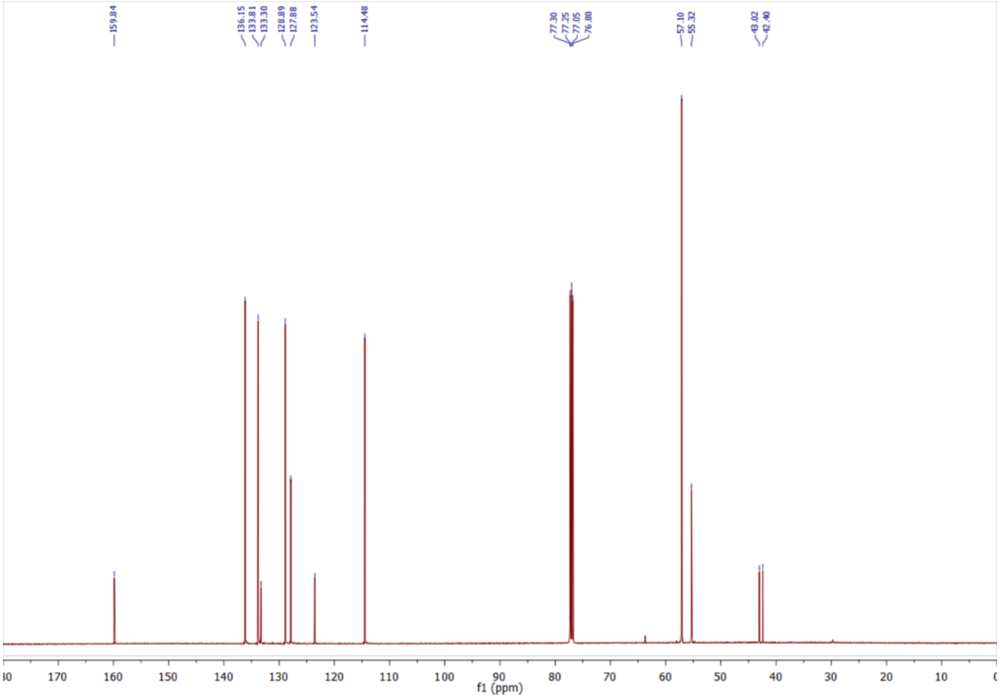

[**17**] 1-Iodo-2-phenylbenzene

^{1}H NMR

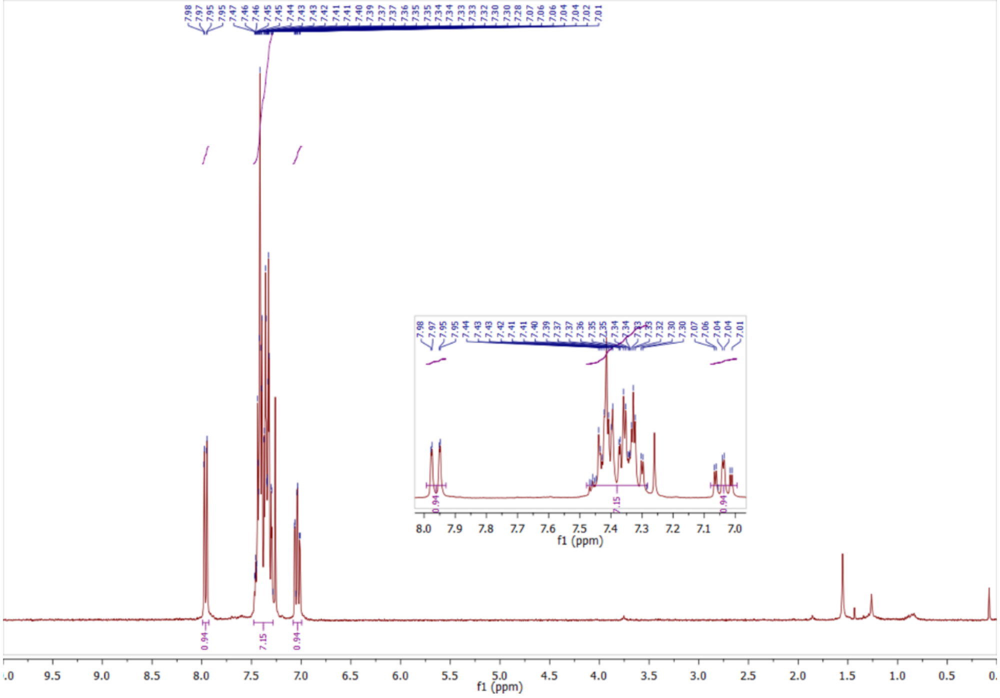

^{13}C NMR

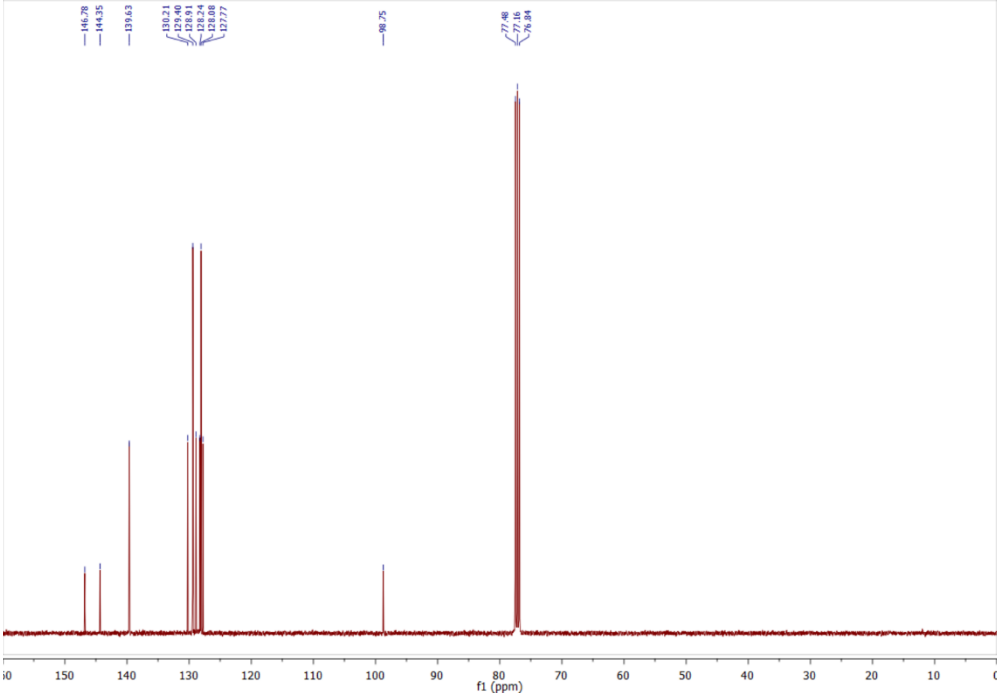

Supplement: File 2 — Spectra. [file Beilstein_J_Org_Chem-15-1172-s002.pdf]
